# Supplementary material for: Challenges and solutions: surveying researchers on what type of community engagement and involvement activities are feasible in low and middle income countries during the COVID-19 pandemic
Source: BMJ Open. 2021 Oct 27;11(10):e052135. doi: 10.1136/bmjopen-2021-052135 (PMC8551745; doi:10.1136/bmjopen-2021-052135)
Supplement: Supplementary data [file bmjopen-2021-052135supp001.pdf]

Appendix 1: Survey questions

Challenges and solutions: What kind of CEI is feasible in LMICs during COVID-19?

As an NIHR global health research fund holder we are inviting you to participate in this survey.

The aim of this survey is to capture what kind of CEI activities are feasible in LMICs under the current challenging global circumstances, including how much CEI activities have changed and been adjusted to the new situation. We would like to know,

- what CEI activities have been unaffected and remain ongoing,
- any challenges faced and
- any potentially new, innovative ways of communication with communities in LMICs.

This survey is purely for learning purposes to share knowledge and expertise across global health networks, both within the NIHR and beyond, and so we can support each other in these challenging times. We hope to highlight efficient communication pathways with communities in LMICs that might be taken forward after this pandemic as general practice.

The survey should take approximately 15-20 minutes to complete. If you are unable to complete the survey in one sitting, your responses will be saved and you can return later to complete it. If you would like to nominate another colleague e.g. the lead for CEI to complete the survey, please forward the survey URL link to them.

Before you consider completing the survey please read the information sheet which was circulated with the email invitation. Thank you.

Your participation

The information you provide is confidential and you will not be identified in the reporting of findings unless you give your permission. General information about you, your role and your institution will be requested to help us analyse the survey results.

Your participation is voluntary. It is an opportunity to help us understand the impact of COVID-19 on CEI in LMIC global health research.

If you would like to complete the survey, please check the box below as your consent to participation. You will not be able to continue to the main survey without doing so.

Thank you for your time and input. It is much appreciated.

If you have any queries, please contact Katie Porter at ceiglobalhealth@nihr.ac.uk.

**1. About you (individual completing the survey) and your associated institution**	
Name	
Job title	
Associated organisation/institution	
Funding body/ies for your research in LMICs	
Broad research area/s	
Please tell us what LMICs you are collaborating with for ongoing and new research	
Are you happy to be contacted for further questions relating to the information you have provided in this survey?	☐Yes, please provide email address:<br><br>☐No

**2. Overview: Ongoing research projects and CEI activities in LMICs**<br>When we refer to ongoing research in this survey, we mean research projects designed and started prior to the COVID-19 pandemic.<br>**If you have no ongoing research projects, please skip ahead to section 6.**	
Are any of your ongoing research projects currently going ahead?	☐Yes<br>☐No

If yes to above:

How many of your projects are currently going ahead with no effect on CEI activities despite COVID-19?	
How many of your projects are currently going ahead, but CEI activities had to be paused due to COVID-19?	
How many of your projects are currently going ahead, but CEI activities had to be amended due to COVID-19?	

**3. Overview: New research projects in LMICs**<br>When we refer to new research in this survey, we mean research projects designed and started after or in response to the onset of the COVID-19 pandemic, to be started within the next few months (whilst COVID-19 is still likely to be affecting research in LMICs)	
Have you planned and/or started any new research projects in LMICs since the start of the COVID-19 pandemic?	☐Yes<br>☐No
If yes, do any of these new research projects have CEI components planned in?	☐Yes<br>☐No
If yes, how many of these projects have CEI components planned in?	

**4. The next 2 sections will ask you to provide more details about the CEI activities with your ongoing and new research projects in LMICs. This is only relevant to you if you have previously stated that you have ongoing or new CEI activities of any kind.**<br><br>**If you have previously selected that ALL your CEI activities had to be paused and/or no new activities were planned, please select "No CEI activities are being done" from the drop down menu to skip to the end of the survey**
☐ I am undertaking CEI activities. I would like to continue the survey<br>☐ No CEI activities are being done

**5. We are interested to capture what kind of CEI activities are feasible under the current circumstances.**<br>**Please provide a summary of what CEI activities are currently ongoing and provide explanation of how they are being carried out. If they are linked to specific research projects only, please fill in those details first.**<br>*(Free text, bullet point answers OK.)*	
Research project	Name:
	LMIC:
	☐Ongoing<br>☐New
CEI activities<br>*(free text)*	

**6. What challenges have you faced in the current climate when carrying out CEI activities and/or planning CEI activities for new studies?**<br>Please provide the main reasons for these challenges.<br>*(free text, bullet points OK)*

**6. Were you able to find any feasible solutions to the challenges faced or new, innovative ways of going about CEI in LMICs during this pandemic?**<br>For example: new, successful ways of communicating with patient/public partners (please provide details of involved groups and LMICs, if so).<br>**Please include lessons learnt and top tips or any promising ideas.**<br>*(free text, bullet points OK)*

**7. Are there any CEI components you designed due to COVID-19 that you might adapt for future research projects?**<br>**If yes, please describe these CEI components and explain why you would continue to use them.**<br>*(free text, bullet points OK)*
