# Supplementary material for: Challenges and solutions: surveying researchers on what type of community engagement and involvement activities are feasible in low and middle income countries during the COVID-19 pandemic
Source: BMJ Open. 2021 Oct 27;11(10):e052135. doi: 10.1136/bmjopen-2021-052135 (PMC8551745; doi:10.1136/bmjopen-2021-052135)
Supplement: Supplementary data [file bmjopen-2021-052135supp002.pdf]

Appendix 2: Round table questions

We invited Network members to talk about their observations and reflections to date of the CEI activities in LMIC research that they are leading on, involved in or are aware of. The aim of this round table was to hear about members' understanding and experience of the impact of COVID-19 on CEI activities in LMICs.

- What CEI activities are currently taking place during the pandemic?
- How have the original planned CEI activities changed as a consequence of COVID-19?
- What is the impact of COVID-19 is on CEI activities in LMIC research?
- What activities have been paused or discontinued?
- What CEI activities have been unaffected?
- How have CEI activities been adapted/changed?
- In summary, what have been the main barriers and challenges?
- How did you overcome these challenges?
- Are the CEI challenges different to the normal ones faced outside of the pandemic?
- Have you explored any new or innovative ways to engage or involve relevant people or communities with both ongoing and new research?
