# Supplementary material for: Challenges and solutions: surveying researchers on what type of community engagement and involvement activities are feasible in low and middle income countries during the COVID-19 pandemic
Source: BMJ Open. 2021 Oct 27;11(10):e052135. doi: 10.1136/bmjopen-2021-052135 (PMC8551745; doi:10.1136/bmjopen-2021-052135)
Supplement: Supplementary data [file bmjopen-2021-052135supp003.pdf]

Appendix 3: Survey participant funding bodies

Funding body	No. of projects
NIHR	27
Medical Research Council (MRC)	8
Wellcome Trust	5
UK Research and Innovation (UKRI)/Research England	3
Economic and Social Research Council (ESRC)	2
Department for International Development (DFID)	2
Gavi The Vaccine Alliance	1
GlaxoSmithKline	1
AstraZeneca	1
Save the Children	1
International AIDS Vaccine Initiative (IAVI)	1
World Bank	1
Norwegian Government	1
National Institutes of Health (NIH)	1
European Union (EU) Research and Innovation programme Horizon 2020	1
European & Developing Countries Clinical Trials Partnership (EDCTP)	1
Bill & Melinda Gates Foundation	1

World Health Organisation (WHO)	1
FAIRMED Foundation	1
European Commission (EC)	1
HIV Vaccine Trials Network (HVTN)	1
The Global Fund	1
Unknown	1
