# Supplementary material for: Challenges and solutions: surveying researchers on what type of community engagement and involvement activities are feasible in low and middle income countries during the COVID-19 pandemic
Source: BMJ Open. 2021 Oct 27;11(10):e052135. doi: 10.1136/bmjopen-2021-052135 (PMC8551745; doi:10.1136/bmjopen-2021-052135)
Supplement: Author's manuscript [file bmjopen-2021-052135.draft_revisions.pdf]

BMJ Open

BMJ Open is committed to open peer review. As part of this commitment we make the peer review history of every article we publish publicly available.

When an article is published we post the peer reviewers' comments and the authors' responses online. We also post the versions of the paper that were used during peer review. These are the versions that the peer review comments apply to.

The versions of the paper that follow are the versions that were submitted during the peer review process. They are not the versions of record or the final published versions. They should not be cited or distributed as the published version of this manuscript.

BMJ Open is an open access journal and the full, final, typeset and author-corrected version of record of the manuscript is available on our site with no access controls, subscription charges or pay-per-view fees (http://bmjopen.bmj.com).

If you have any questions on BMJ Open's open peer review process please email info.bmjopen@bmj.com

BMJ Open

Challenges and solutions: What kind of Community Engagement and Involvement (CEI) is feasible in low and middle income countries (LMICs) during COVID-19?

Journal:	*BMJ Open*
Manuscript ID	bmjopen-2021-052135
Article Type:	Original research
Date Submitted by the Author:	08-Apr-2021

Complete List of Authors:	Kroese, Karolin; University of Birmingham<br>Porter, Katie; University of Southampton<br>Surridge, Heidi; University of Southampton<br>Tembo, Doreen; University of Southampton

Keywords:	COVID-19, STATISTICS & RESEARCH METHODS, INFECTIOUS DISEASES

SCHOLARONE™
Manuscripts

I, the Submitting Author has the right to grant and does grant on behalf of all authors of the Work (as defined in the below author licence), an exclusive licence and/or a non-exclusive licence for contributions from authors who are: i) UK Crown employees; ii) where BMJ has agreed a CC-BY licence shall apply, and/or iii) in accordance with the terms applicable for US Federal Government officers or employees acting as part of their official duties; on a worldwide, perpetual, irrevocable, royalty-free basis to BMJ Publishing Group Ltd ("BMJ") its licensees and where the relevant Journal is co-owned by BMJ to the co-owners of the Journal, to publish the Work in this journal and any other BMJ products and to exploit all rights, as set out in our licence.

The Submitting Author accepts and understands that any supply made under these terms is made by BMJ to the Submitting Author unless you are acting as an employee on behalf of your employer or a postgraduate student of an affiliated institution which is paying any applicable article publishing charge ("APC") for Open Access articles. Where the Submitting Author wishes to make the Work available on an Open Access basis (and intends to pay the relevant APC), the terms of reuse of such Open Access shall be governed by a Creative Commons licence – details of these licences and which Creative Commons licence will apply to this Work are set out in our licence referred to above.

Other than as permitted in any relevant BMJ Author's Self Archiving Policies, I confirm this Work has not been accepted for publication elsewhere, is not being considered for publication elsewhere and does not duplicate material already published. I confirm all authors consent to publication of this Work and authorise the granting of this licence.

For peer review only

Challenges and solutions: What kind of Community Engagement and Involvement (CEI) is feasible in low and middle income countries (LMICs) during COVID-19?

Authors: Dr Karolin Kroese [1], Katie Porter [2], Heidi Surridge [2], Dr Doreen Tembo [2]

Affiliations:

1. NIHR Global Health Research Unit on Global Surgery, University of Birmingham, Birmingham, UK
2. Wessex Institute, Faculty of Medicine, University of Southampton, Southampton, UK

Correspondence: Katie Porter, katie.porter@nihr.ac.uk

Word count (excluding title page, abstract, references, figures and tables): 4991 words

For peer review only

Abstract

Objectives: Measures to limit the spread of infection during the COVID-19 global pandemic have made engaging and involving members of the community in global health research more challenging. This research aimed to explore how global health researchers adapted to the imposed pandemic measures in low and middle income countries (LMICs) and how they overcame challenges and barriers to effective community engagement and involvement (CEI).

Design: A qualitative two-stage mixed methods study involving an online survey and a virtual round table.

Setting: The survey and round table were completed online. Participants based at various institutions and organisations across the UK and in LMICs.

Participants: Of 53 participants, 43 were LMIC or UK-based global health researchers and/or CEI professionals, and 10 worked for the National Institute for Health Research or UK Government's Department of Health and Social Care.

Outcome measures: There were no primary or secondary outcome measures due to the qualitative nature of the study. Outcomes will be presented as overall common emergent themes.

Results: Pandemic control measures forced the majority of researchers to stop, or amend their planned CEI activities. Most face-to-face CEI activities were then replaced with remote methods, such as virtual communication platforms. Virtual engagement enabled researchers to maintain already established relationships with community members, but was less effective when developing new relationships or addressing challenges around the inclusion of marginalised community groups.

Conclusions: COVID-19 has highlighted the need for contingency planning and flexibility in CEI. The re-designing and adopting of remote methods has come with both advantages and disadvantages, and required new skills, access to technology, funding, reliable services and enthusiasm from stakeholders including community members. The methods suggested have the potential to augment or substitute

For peer review only - http://bmjopen.bmj.com/site/about/guidelines.xhtml

previously preferred CEI activities. The effectiveness and impact of these remote CEI activities need to be assessed.

Article summary: Strengths and limitations of this study

- This study used a two-stage mixed method evaluation using an online survey and an international round table to capture the impact of the COVID-19 pandemic on community engagement and involvement activities.
- The participants represent a broad variety of LMICs across multiple continents, therefore providing a good overview of CEI activities across the board.
- The level of seniority and expertise of participants is varied, ensuring an accurate representation of CEI adaptation across the global health research network.
- Data was triangulated and coded to generate common emergent themes from the qualitative data to understand the main challenges and barriers research teams have faced, and how their community engagement and involvement activities have been adapted in global health research.
- Due to the sample size and timeframe when data was collected, this study only provides a snapshot of the community engagement and involvement activities being done in global health research early on in the pandemic (June/July 2020), but it provides an opportunity for shared learning and contingency planning in a unique situation.

Introduction

The development and expansion of the UK National Institute for Health Research (NIHR) and other UK-based funding bodies into the Global Health arena has necessitated a rethink of what is known as patient and public involvement (PPI) in the UK. UK-based global health researchers had to explore ways to involve community members in low and middle income countries (LMICs) where their research is taking place, using paradigms that are feasible, accepted and effective in a local context. In global health research, the involvement of public members is internationally more commonly referred to as community engagement [1], and within NIHR's global health research community as community engagement and involvement (CEI).

While there are some variations in the definition of CEI, the one used by the US Centres for Disease Control and Prevention summarises the essence of community engagement as: "…the process of working collaboratively with and through groups of people affiliated by geographic proximity, special interest, or similar situations to address issues affecting the well-being of those people" [2]. Community can broadly be defined as a minimum social unit who have a stake in the proposed research. This could include individuals, groups, organisations, government bodies, peer-groups or social networks, and those who can influence or who may be affected by the research [3]. Community involvement can therefore be defined as the community actively participating or being involved in any aspect of the research cycle [4].

For peer review only - http://bmjopen.bmj.com/site/about/guidelines.xhtml

Carrying out meaningful CEI within research is important as it reduces the potential for exploitation of communities and facilitates the implementation of health research [4]. Furthermore, involving communities in LMICs in health research funded by high income countries (HICs) serves to address possible ethical concerns and cultural differences such as distrust and concerns about scientific colonialism [5]. If done properly, e.g. following the UNICEF standards [1], CEI can help ensure that benefits of research can be utilised by those in need, build on local capacity and support a true culture change in low resource settings by empowering people at grassroots level.

As COVID-19 developed into a global pandemic and countries went into lockdown, engaging and involving members of the public in research became far more challenging. In this paper we define 'lockdown' as large-scale physical distancing measures and movement restrictions [6], however we are aware that this term is specific to a UK context and not necessarily used globally. Similarly, 'post-lockdown periods' are not a unified, global point in time, and are varied in both severity and timeframes.

Solutions to involving the public in research during lockdown in the UK rapidly evolved around online communication platforms, however, this may not be feasible or poses additional challenges in LMICs. In LMICs, CEI has focused heavily on face-to-face communication due to limited access to technology and the internet. Traditionally face-to-face communication has been relied upon as a direct way to overcome barriers to involvement and is a cultural preference in some countries [7].

This project focused on investigating how CEI in low-resource settings developed and adapted to the restrictions imposed as a response to the COVID-19 pandemic. We aimed to understand the impact the restrictions had on CEI in LMICs and the main challenges researchers had to overcome. We highlight how CEI is being redesigned to address some of these barriers, explore the limitations with these solutions and discuss the implications for CEI.

Methods

We used a two-stage mixed method evaluation using an online survey and an international round table. Data was collected across June to early July 2020. Three of the authors work for the NIHR, but are not directly involved in the commissioning process, and so were able to use their networks to contact and involve NIHR-funded awardees and the NIHR CEI Global Health Advisory Network in this research.

Survey

An online survey was designed to capture the impact of COVID-19 on CEI activities, the main challenges and barriers research teams have faced, and how CEI activities have been adapted (see appendix 1). The survey captured quantitative data on demographics of participants and their research, as well as qualitative free text data on their CEI pandemic experiences. Members of the public and NIHR CEI professionals reviewed the survey and contributed to its development.

For peer review only - http://bmjopen.bmj.com/site/about/guidelines.xhtml

The sampling used a combination of purposive and snowballing techniques [8]. The survey link, an invitation email and information sheet, were sent to all NIHR funded (and thus UK Aid funded) Global Health researchers and infrastructure awardees (n=70). The survey invitation was also posted on the Mesh Community Engagement Network website to access non-NIHR funded researchers. NIHR colleagues and networks shared the survey invitation with their own networks in the Global Health Research Community.

Round table

A round table discussion with the NIHR CEI Global Health Advisory Network [9] exploring the same topic was hosted as part of this research project. The questions asked at the round table can be found in appendix 2. 12 LMIC and UK-based global health researchers took part in the facilitated discussion in addition to 10 NIHR and UK Government's Department of Health and Social Care (DHSC) staff members.

Data analysis

The data from the survey and round table was triangulated and three individual members of the team used open coding to subsequently generate common emergent themes from the qualitative data, which are described in the results section. Analysis was supported by Microsoft Excel and NVivo.

Patient and Public Involvement

As this research aimed to capture the researcher's perspective, patients and community members were not directly involved. The collected data describes how research teams are engaging and involving community members in the research process. However, relevant stakeholders, such as experienced NIHR patient and public involvement managers, were asked to review the questions and trial the online survey tool for usability and clarity prior to it being circulated.

Results

Survey participant demographics

Thirty-one (31) surveys were returned, most of which were submitted by professors (8, 25%) or project/programme managers (7, 22.5%) based at UK Universities (22, 70%). Of these 31, 27 (87%) were in receipt of NIHR funding, but many reported support from other funders (see appendix 3). Most participants reported on multiple research projects in different LMICs, in thematically diverse research areas. Participants' research was being conducted mainly on the African continent (61 out of 116 individual projects for this question, 53%), followed by Asia (39 individual projects, 33%) and South America (10 individual projects, 9%).

Round table participant demographics

Twenty-two (22) people took part in the round table discussion: 7 (32%) were LMIC-based global health researchers, 5 (23%) UK-based global health researchers, and 10 (45%) UK-based NIHR and DHSC staff members. Of the 7 LMIC-based researchers, 4 (57%) lived and worked in sub-Saharan Africa, 3 (43%) lived and worked in South and Southeast Asia. Of the NIHR and DHSC staff members, 4 (25%) worked within a Global Health Research team, and 6 (75%) had roles within PPI/CEI teams.

For peer review only

Impact of COVID-19 restrictions on CEI activities in LMIC based research

All respondents stated that initially all their studies included CEI components, which ranged from consultative activities like community advisory boards/groups and focus groups to more involved activities such as co-production of study materials, or members of communities undertaking data collection.

Due to COVID-19, 83% of survey participants' planned CEI activities had to be amended or paused. Out of the 31 survey participants, 28 (90%) stated that their studies or aspects of them are currently going ahead despite the pandemic. However, only five of these 28 participants (17%) stated that their CEI activities with one or multiple projects are going ahead as planned. 24 out of the 28 participants (86%) with ongoing projects had to pause some or all of their CEI activities due to the pandemic, and 22 out of 28 (79%) participants stated that some or all of their CEI activities had to be amended, with there being overlap between the amended and paused answers. 14 out of all 31 participants (45%) stated that they are currently planning new LMIC-based research projects, with 11 (78%) having CEI components planned in.

Reasons for halting CEI activities

Restriction of face-to-face interaction

During country-specific periods of lockdown, which included non-essential travel restrictions and social distancing measures, all face-to-face CEI activities had to be paused. Restrictions meant that researchers were unable to travel to different geographical areas, especially remote locations, for research purposes, such as providing interventions or collecting data.

> *"CEI which involved movement of researchers from one area to another [...] were also halted to avoid the spread of COVID-19. This has had a serious negative impact on CEI [...] the expected beneficiaries did not benefit anything from the CEI community dementia awareness raising planned activities." (Survey respondent 25)*

Local engagement activities throughout the research process were reported by many as involving regular face-to-face communication with researchers, multi-level government and organisational stakeholders, as well as community members. In Africa, many engagement activities are carried out face-to-face with community leaders and other representative bodies, as a culturally well-respected form of communication (*round table member 2*).

> *"We had planned to develop all materials through co-design and to undertake a series of focus groups for this purpose within India. However, we have been unable to complete this." (Survey respondent 4)*

For peer review only - http://bmjopen.bmj.com/site/about/guidelines.xhtml

"The team in Nepal were already heavily involved in a number of community awareness campaigns, using radio, tv, local schools, women's cooperatives etc. Due to Covid-19 these were all cancelled." (Survey respondent 3)

Diversion of resources

In some countries, governments decided that all non-essential research should be halted. Others found that their research partners were diverted to other pandemic-related activities or research.

"In two of the three countries the ethical and regulatory authorities have ordered that all "non-essential" research activities are stopped. We have been able to continue our interventional clinical trials as they are deemed to be of direct importance to patient care, but the CEI activities are viewed as non-essential." (Survey respondent 23)

"Due to the pandemic, and our respiratory specialty, our teams have been pulled to the front-line- while in key stages of completing our programme's milestones!" (Survey respondent 7)

CEI solutions to pandemic measures

Some researchers re-designed their CEI activities during lockdown to be able to deliver at least certain objectives and to maintain contact with community stakeholders.

Remote methods of CEI

Due to pandemic restrictions, online methods replaced planned face-to-face activities. These have been highlighted as positive alternatives for one-way communication, such as sharing documents or videos for training and awareness raising. A key benefit is that resources can be shared widely and repeatedly with minimal effort, cost and time.

"We now have a video recording of all our training and don't hand out paper manuals... Everyone receives a copy of the training on a USB and is free to revisit it whenever they need to." (Survey respondent 10)

Researchers in the Occupied Palestinian Territories found that large scale online campaigns to raise awareness of burns studies has proven effective, but where materials could not be distributed online, they planned to combine their CEI activities, in this case health research awareness raising materials, with ongoing COVID-19 initiatives.

"Pamphlets and materials were prepared for a house-to-house community awareness campaign before COVID-19 lockdown. They [Ethiopia research team] are working with the Ministry of Health to see if they can combine their work on spreading COVID-19 messages which will be house-to-house [...] with burn prevention messages." (Survey respondent 3)

For peer review only - http://bmjopen.bmj.com/site/about/guidelines.xhtml

For two-way communication, researchers quickly adapted to using video calls (Zoom, Skype, GoToMeeting), phone, text, WhatsApp, WeChat, and similar apps. Online workshops and questionnaires were also introduced as participants adapted to the restrictions. Researchers pointed out that WhatsApp groups and Zoom meetings are helpful communication tools across borders and for communication between meetings. Some are keen to maintain communication via these platforms after country-specific pandemic restrictions are lifted, in addition to face-to-face meetings, to ensure continuity of contact with community stakeholders.

> *"As the groups have been engaged from the initial stages of the research they are keen to continue and provide input into the developing projects […] WhatsApp groups are the most useful method of communication for the CEI groups in the countries in which we work. The groups used this method between meetings, but also complete group meetings now using this platform." (Survey respondent 15)*

> *"We have been pleasantly surprised by the relatively good internet access available to our researchers in Nepal who have been working from home. This is encouraging as it suggests that domestic internet access is better and more widespread than we initially imagined and may mean that some CEI activity is potentially feasible." (Survey respondent 17)*

Online communication methods were well received by young people in some LMICs. For example, in Nepal, Facebook groups were created to help young men discuss their mental health and wellbeing during lockdown (*round table member 6*).

Planning of post-lockdown CEI activities

The enforced period of restrictions allowed researchers to develop and adapt research and CEI plans for future post-pandemic research projects, whilst recognising that flexibility may be needed long-term.

> *"All activity has been delayed due to COVID-19 but we have been able to do all the planning and will be ready to do face-to-face work once restrictions in Uganda and Tanzania are over. Things like filming and getting groups of patients together has not been possible due to restrictions within the country." (Survey respondent 5)*

For peer review only

Challenges with adapted CEI activities

Small group meetings post lockdown

In some countries, participants reported that small group gatherings were possible as restrictions began to ease. However, the extra precautions of personal protective equipment (PPE) and smaller community group meetings has higher resource implications for CEI.

> *"The inability to hold massive community meetings at halls during this time is really challenging [...] Currently, we have broken down our scale to less than 30 per meeting which means more meetings and outings than preferred." (Survey respondent 10)*

A research team also reported that on return to the community, post-lockdown, wearing the necessary facemasks and PPE resulted in caution and mistrust as they were not easily identifiable (*round table member 7*).

Community and stakeholder availability and priorities

Research partners in LMICs have found themselves diverted to the pandemic response, whilst others have reduced CEI in order to keep their research sustainable. Furthermore, the pandemic restrictions resulted in community members having to prioritise basic needs of food, work and care for their families over engaging with researchers.

> *"As our partners are all clinicians with a focus on respiratory care, many of them are being pulled into the front line. Because of their respiratory specialty, and their primary care practice, they may also be involved for a longer term than researchers with other fields of interest." (Survey respondent 7)*

> *"Fear from getting infected so they refuse participation in research. Lockdown leaves them with few hours to respond to researchers, their priority is their children, food etc." (Survey respondent 11)*

Inclusivity: Access to, and connectivity with, remote technology

Whilst remote means of communication have been the main CEI method during COVID-19, it has not been without its challenges and limitations. The use of digital platforms all rely on the individuals having access, funds and ability to use the technology and the internet.

In India, access to a smartphone was common but many could only access the internet on their devices at work, where free WiFi was provided (*survey respondent 4*). One research team working in sub-Saharan Africa noted that WhatsApp has enabled researchers to continue their CEI as even though computer access is rare, mobile phone access is common (*survey respondent 15*). Others working in Liberia and Nepal reported no or limited access to computer or mobile devices. Where devices were available, participants reported that larger-scale remote meetings, workshops or

For peer review only - http://bmjopen.bmj.com/site/about/guidelines.xhtml

training were problematic due to unreliable internet connections. To surmount some of these issues respondents suggested that research teams could provide data allowances and technology for community stakeholders already involved with their research to ensure ongoing communication. Obviously, this has cost implications and may need to be costed into funding applications under CEI budget.

> *"The challenge of poor Internet connectivity often frustrated seamless virtual meetings with colleagues and stakeholders. We had to spend more funds purchasing alternative data service provider modems, and increased budget providing data for team members working from home." (Survey respondent 31)*

> *"Communication is often challenging as a lot of the groups that we engage with have limited access to technology platforms- the digital divide." (Liberia - Survey respondent 27)*

Teams who set up virtual groups using text-based platforms, such as WhatsApp, faced language and literacy barriers. One research group reported using voice notes as a way around this (*survey respondent 24*). An increased use of online communication and use of smartphones for CEI activities also highlights data privacy and safeguarding issues. Participants noted that being able to see everyone's numbers on WhatsApp raised safeguarding concerns.

Remote communication was also thought to further marginalise some groups, for example in a study in Pakistan, husbands were often gatekeepers to women's phone access (*survey respondent 12*). Another team working with religious minority groups and in slum settlements found that many people, especially women and older people, had no internet access (*round table member 7*).

> *"Unable to have face to face contact since lockdown due to COVID-19 so using telephone but this can be difficult for some groups such as women in Pakistan who may only have access to their husband's telephone[…] Could miss some of the most vulnerable people in the community by only having telephone communication." (Survey respondent 12)*

Many study participants reported that online and digital communication for CEI activities highlighted the social and economic inequalities within and between countries.

Quality of relationships built remotely

Some participants referred to remote communication as "second best" - an interim measure with concerns based on quality. Telephone and virtual meeting conversations were described as shorter, formal and less natural than face-to-face interactions. They were deemed tolerable for established relationships, but not ideal for making crucial stakeholder connections and establishing new relationships with community members. Respondents therefore tended to focus on maintaining and developing relationships that were established before lockdown.

For peer review only - http://bmjopen.bmj.com/site/about/guidelines.xhtml

"Face to face communication is highly valued in Nepali society. Taking the trouble to visit someone in person demonstrates that you value them and their opinion, and is essential for building relationships." (Nepal – Survey respondent 17)

"We have also used online Zoom webinars, which are useful in engaging people and groups, however, compared to face-to-face meetings, they lack the networking and 'connection' which face-to-face conferences and meetings have; (the personal connection, when speaking to colleagues at dinners or over coffee), this is very personal and valuable." (China – Survey respondent 7)

Some research teams have reverted to online surveys and posting information, where previously these engagements would have been conducted face-to-face and hence interactively.

For peer review only

Discussion

Impact of COVID-19 on CEI

CEI activities are important for researchers in building and maintaining trust with local communities [10]. The authoritarian nature of strict lockdowns and lack of information on COVID-19 in some LMICs created fear and distrust among communities. As lockdown restrictions ease globally, researchers need to consider elevated levels of suspicion, especially among new contacts. Issues may include fear of strangers bringing the virus into communities, or communities being wary of researchers who are wearing PPE as they are not easily identifiable.

Lockdown restrictions have exacerbated poverty in already impoverished areas, because people cannot go to work and receive income [11]. People have been focused on meeting basic needs and looking after their own families' wellbeing and health rather than getting involved in research engagement activities. As research continues, project teams will need to consider the ethics of continuing CEI activities in relation to the needs of the community, especially as this pattern may continue as communities face prolonged impact of pandemic restrictions.

Impact of COVID-19 on research

The quality and impact of the amended CEI activities and impact on the research they support has yet to be investigated, especially when considering the limitations to remote CEI activities. It has been reported that CEI adaptations to pandemic measures have ultimately been inequitable and the needs of resource-poor and underrepresented communities were given little consideration [12]. The rapid response of many COVID-19 research projects have caused some to abandon regular engagement processes and focus on the voice of "professionals" [13]. In emergency pandemic situations, we need to ensure that the voice of the community and public should not be lost [14].

Reduced or poorer quality CEI may affect the outcomes of a research study, it's impact [1] and potentially opportunities for future funding. A feasible solution to further include relevant

For peer review only - http://bmjopen.bmj.com/site/about/guidelines.xhtml

community stakeholders suggested by LMIC researchers in this study is to utilise grassroots organisations and mobilise local civil society groups, which has been an effective way during COVID-19 to reach communities and a faster route for communication [15, 16].

Pros and cons of virtual engagement

Good working relationships with community stakeholders are essential to ensure the value and quality of research [17, 18], however trusted relationships take time to develop. Remote two-way communication has worked better for those researchers who have established relationships with community stakeholders. Building and maintaining meaningful relationships with the community are an important component of CEI [19], and this study has shown that pre-pandemic relationships may be sustained in spite of the lack of face-to-face engagement.

Text and voice messaging apps were reported as useful in maintaining continuity of relationships during the pandemic, and were already a well-established communication method. A combination of voice/text messaging and video meetings can provide an alternative to face-to-face meetings. Virtual meetings can also provide a solution when there is no pre-planned funding for travelling to or hosting meetings with community members and LMIC-based researchers.

Collaboration and co-production of research relies on the development of trusting relationships [6, 10, 19]. Even though online platforms allow researchers and healthcare professionals to stay in touch with members of the community, there are certain limitations to this kind of engagement. These virtual CEI methods have been less effective in reaching out and engaging new communities, especially isolated vulnerable groups [20].

Inclusivity concerns

The digital divide

Often, community members in LMICs have no or limited access to devices or the internet, and when they do, internet connection may be unstable or confined to certain areas. Thus, lockdown restrictions have meant that researchers struggled to reliably reach some community members. This means the most marginalised are even more likely to be excluded. This is an issue in HICs as well as LMICs, highlighting the need for non-technology based engagement [21]. Although participants suggested that researchers can pay for data allowances and technology, this raises questions around whether funders would be willing to cover these costs.

Some digital video software might provide a solution for people who have access to a phone, but no internet connection, as people can dial into meetings via phone calls. It could also be more cost-effective, accessible and increase reach and diversity [22, 23]. However, for community members to use

For peer review only - http://bmjopen.bmj.com/site/about/guidelines.xhtml

the application and feel confident to contribute in meetings, training might be required. Depending on the capacity of the research team, this may pose an issue when trying to involve large numbers of community members.

Age may also be a factor as young people, particularly those in developing countries, who have access to gadgets or the internet may already be comfortable using these methods of communication [24, 25, 26]. Thus engagement with this age group via online platforms may be easier for researchers compared to engaging older age-groups [7].

Technology also raises concerns of data privacy in LMICs and HICs alike and is a concern in PPI/CEI, as well as telemedicine [27, 28]. However, data privacy is not a new concern especially in healthcare [28]. An adequate solution for data privacy is yet to be found [28].

Close-knit communities often rely on 'word of mouth', where information is passed verbally, in-person to members of the community who do not have access to technology [29]. This approach should not be encouraged in pandemic situations and is prone to perpetuating inaccuracies through onward translation.

Language and literacy barriers

Messaging apps were used for ongoing communication with CEI groups, but participants reported language and literacy barriers. Members of a messaging group may speak different dialects or languages depending on where they are based within the country - or across countries. The reach across borders via virtual platforms, poses the challenge of finding a common language. Where face-to-face engagement would usually take place with skilled translators, rapid and accurate translation is not an easy option in live virtual chats and meetings [30].

Marginalised communities

Participants reported that a common way to reach community members is via gatekeepers, who are often health workers or community leaders (for example, faith leaders or community elders). Accessing marginalised groups via gatekeepers is an established method for reaching a certain cohort of people based on, for example, location or faith [31]. However, participants noted that there can be limitations when accessing these communities via gatekeepers. This research found that in Pakistan, in some communities, women do not have access to personal technological devices, so the involvement of women in the participant's research depended on the agreement of their husbands or male relatives.

Another factor is the influence and power that gatekeepers often have over their communities. Some gatekeepers may be resistant to or sceptical of change, and may use their influence over the community to sway their opinions [32]. Restrictions on face-to-face meetings mean that this issue may

For peer review only - http://bmjopen.bmj.com/site/about/guidelines.xhtml

be enhanced, due to researchers relying fully on gatekeepers communicating potentially complex studies to communities - potentially influencing audiences with biased views.

Limitations

The data for this research has come from a relatively small sample and was collected relatively soon after COVID-19 had become a global pandemic, and so provides only a snapshot of the CEI activities being done in global health research. However, the authors saw this as an opportunity for shared learning in a unique situation and recommend that further research is done looking at the long-term impact of the COVID-19 pandemic on CEI activities.

Conclusion

CEI adaptations were made in a fast changing environment. There is much learning to be had for future similar localised and global situations, as well as during the ongoing COVID-19 pandemic. As countries gradually move towards a "new normal", the way CEI is done may have changed forever as many researchers have experienced benefits from their adapted CEI activities.

Below are recommendations that research teams and funders should consider as research continues in uncertain pandemic (or communicable disease) situations.

Use time effectively

Time saved due to travel restrictions can be used to plan and adapt future CEI activities. This time can also be used to develop the skills of the research team and community members, in particular technology and online system skills.

Share networks and resources

Researchers have been struggling to engage new communities during the COVID-19 pandemic. Moving forward, research teams could utilise the expertise, networks, and resources of others. For example, linking with other research teams to share and engage with relevant networks, or working with LMIC-based grassroot organisations and government initiatives to reach and engage communities. Existing relationships can then be built upon which may also enable rapid response mechanisms, especially with marginalised communities.

Promote acceptance and adoption of online technologies

Generally, remote platforms for CEI activities have differed from what was originally planned, and the pandemic has required researchers to be creative. Research teams need to think flexibly about what is feasible, and how various technologies and online tools can be used in the longer term. The sudden onset and fluctuating incidence of this pandemic and subsequent restrictions means ongoing uncertainty and contingency plans for CEI should be in place. Like researchers, funding bodies could consider room for flexibility for CEI activities proposed by the applicant, e.g. welcoming alternative

For peer review only - http://bmjopen.bmj.com/site/about/guidelines.xhtml

and multiple scenario CEI activities for proposed research projects, and allowing for re-allocation of funds within the budget.

Research teams, as well as funders, will need to maximise their knowledge and creativity with virtual engagement whilst accepting that there may be limitations. Maintaining trust and relationships with community members is now more important than ever to ensure that they do not feel abandoned in times of crisis, to make research relevant and beneficial and to allow for involvement of key stakeholders when designing and implementing new studies remotely (for example, rapid response for COVID-19 studies). The use of digital tools is a way to do this, albeit perceived as not as inclusive or with the same depth as face-to-face communication. This may change with improving knowledge, familiarity and access, and younger people may often prefer the speed and convenience of digital engagement. Remote communication also ensures ongoing transparency by keeping community stakeholders informed of research progress and providing opportunities to contribute at all stages. However, the impact on the quality of CEI undertaken remotely or via mixed approaches needs to be investigated.

Adapt to the changing needs of the community

A pandemic situation re-emphasizes the community's priorities and in low-resource settings in particular, basic needs of access to food, work, health and safety are prioritised over involvement with research. As a way to keep communities engaged, researchers could explore options to partner with others delivering community health interventions/awareness raising activities, such as COVID-19 vaccination programmes. These grassroots community workers, if adequately resourced, may be able to deliver wider CEI activities alongside their own interventions. Funders also need to understand shifting community needs and priorities and potentially allow for re-prioritisation in projects in such circumstances.

Develop contingency CEI plans to tackle the unexpected

There may need to be contingency plans in place for managing CEI in areas with high prevalence of unpredictable communicable diseases. These contingency plans would need to include funding, access and support in the alternative means of communication and activities for all stakeholders and community members involved in CEI. This is naturally an issue that reaches beyond CEI and research alone.

Declarations

Contributorship statement: KK, HS and KP contributed equally to the design and implementation of the research, to the analysis of the results and to the writing of the manuscript. DT contributed to,

For peer review only - http://bmjopen.bmj.com/site/about/guidelines.xhtml

and commented on, this paper. The corresponding author attests that all listed authors meet authorship criteria and that no others meeting the criteria have been omitted.

Acknowledgements: We thank the UK National Institute for Health Research (NIHR) and Department of Health and Social Care staff who provided input into the paper based on their personal views. These included Gary Hickey (senior research manager, community engagement and involvement, UK NIHR), Aaronjay Tidball (global health research programme officer, science, research and evidence directorate, DHSC), Charlotte Seeley-Musgrave (global health research officer, global health research partnerships, DHSC), Patrick Wilson (head of global health communications and stakeholder engagement, UK NIHR), Sarah Puddicombe (assistant director, global health research, UK NIHR) and Jon Cole (assistant director, public involvement and external engagement, UK NIHR). We are also grateful to the NIHR CEI Global Health Advisory Network members who participated in the round table discussion.

Competing interests: We have read and understood BMJ's policy on declaration of interests and declare no conflicts of interest.

Ethics approval: We obtained full ethical approval for the research from the University of Southampton's Faculty of Medicine Ethics Committee (no. 53873).

Data sharing: All data relevant to the study are included in the article or uploaded as supplementary information. Additional data that support the findings of this study (free text data) are available from the corresponding author, Katie Porter, upon request.

Funding: This research was funded internally by the National Institute of Health Research (NIHR). Award/Grant number is not applicable.

References

1. Bedson J, Abramowitz S. Minimum Quality Standards and Indicators for Community Engagement. [Internet] UNICEF; 2020. Available from: https://www.unicef.org/mena/reports/community-engagement-standards [Accessed 9th November 2020]
2. McCloskey, DJ., McDonald, MA., Cook, J. et al. Chapter 1 Community Engagement: Definitions and Organizing Concepts from the Literature. In: Principles of Community Engagement. 2nd ed. Clinical and Translational Science Awards Consortium Community Engagement Key Function Committee Task Force on the Principles of Community Engagement; 2011: 3-41 [Internet] Available from: https://www.atsdr.cdc.gov/communityengagement/pce_intro.html [Accessed 9th November 2020]
3. Council for International Organizations of Medical Sciences (CIOMS). International Ethical Guidelines for Health-related Research Involving Humans. 4th ed. Geneva; 2016: 25-27 [Internet] Available from: https://cioms.ch/wp-content/uploads/2017/01/WEB-CIOMS-EthicalGuidelines.pdf [Accessed 9th November 2020]
4. Musesengwa, R., Chimbari, M.J. Community engagement practices in Southern Africa: Review and thematic synthesis of studies done in Botswana, Zimbabwe and South Africa. Acta Trop 2017; 175: 20-30. Available from: http://dx.doi.org/10.1016/j.actatropica.2016.03.021

For peer review only - http://bmjopen.bmj.com/site/about/guidelines.xhtml

5. King, KF., Kolopack, P., Merritt, MW., and Lavery, JV. Community engagement and the human infrastructure of global health research. BMC Medical Ethics 2014; 15(84). Available from: https://doi.org/10.1186/1472-6939-15-84
6. WHO. Coronavirus Disease (COVID-19): Herd Immunity, Lockdowns And COVID-19. [online] 2020. Available at: https://www.who.int/news-room/q-a-detail/herd-immunity-lockdowns-and-covid-19 [Accessed 18 January 2021]
7. Tindana PO, De Vries J and Kamuya D. Ethical challenges in community engagement practices in research during the COVID-19 pandemic in Africa [version 1; peer review: 1 approved, 1 approved with reservations]. AAS Open Res 2020, 3:23. Available from: doi.org/10.12688/aasopenres.13084.1
8. Streeton, R, Cooker, M, and Campbell J. Researching the researchers: Using a snowballing technique. Nurse Researcher (through 2013). 2004;12(1):35-46. Available from: https://search.proquest.com/scholarly-journals/researching-researchers-using-snowballing/docview/200839960/se-2?accountid=13963
9. Mesh. 2021. NIHR Global Health CEI Advisory Network. Mesh. [online] Available at: <https://mesh.tghn.org/programme-hubs/nihr-global-health-ce-involvement/> [Accessed 1 February 2021].
10. Folayan, MO, Brown, B, Haire, B, Babalola, CP, Ndembi, N. Considerations for stakeholder engagement and COVID-19 related clinical trials' conduct in sub-Saharan Africa. Developing World Bioeth. 2020; 00: 1– 7. Available from: https://doi.org/10.1111/dewb.12283
11. Dahab, M, van Zandvoort, K, Flasche, S et al. COVID-19 control in low-income settings and displaced populations: what can realistically be done? Confl Health 2020; 14(54). Available from: https://doi.org/10.1186/s13031-020-00296-8
12. Cash R and Patel V. Has COVID-19 subverted global health? The Lancet. 2020; 395(10238): 1687-1688. Available from: https://doi.org/10.1016/S0140-6736(20)31089-8
13. Murphy E., Tierney E., Shé, Éidín N N., Killilea M., Donaghey C., Daly A., Roche M., Mac Loughlin D., Dinneen S.F., Panel P.I. COVID-19: Public and patient involvement, now more than ever. HRB Open Res. 2020;3:35. Available from: doi: 10.12688/hrbopenres.13067.1
14. Marston, C, Renedo, A, and Miles S. Community participation is crucial in a pandemic. The Lancet. 2020;395(10238):1676-1678. Available from: https://doi.org/10.1016/S0140-6736(20)31054-0
15. Ratneswaren, A. The I in COVID: The importance of community and patient involvement in COVID-19 research. Clinical Medicine Journal. 2020; 20(4):e120-122. Available from: doi.org/10.7861/clinmed.2020-0173
16. Ekzayez A, al-Khalil M, Jasiem M, et al. COVID-19 response in northwest Syria: innovation and community engagement in a complex conflict. Journal of Public Health 2020; 42(3): 504-509, Available from: https://doi.org/10.1093/pubmed/fdaa068
17. Ryan MJ, Giles-Vernick T & Graham JE. Technologies of trust in epidemic response: openness, reflexivity and accountability during the 2014-2016 Ebola outbreak in West Africa. BMJ Global Health. 2019;4:e001272. Available from: http://dx.doi.org/10.1136/bmjgh-2018-001272
18. Woolf SH, Zimmerman E, Haley A & Krist AH. Authentic engagement of patients and communities can transform research, practice, and policy. Health Affairs. 2016;35(4):590-594. Available from: https://www.healthaffairs.org/doi/pdf/10.1377/hlthaff.2015.1512

For peer review only - http://bmjopen.bmj.com/site/about/guidelines.xhtml

19. Wilson, P, Mathie, E, Keenan, J, et al. ReseArch with Patient and Public invOlvement: a RealisT evaluation – the RAPPORT study. Southampton (UK): NIHR Journals Library; 2015 Sep. (Health Services and Delivery Research, No. 3.38.) Chapter 7, Discussion. Available from: https://www.ncbi.nlm.nih.gov/books/NBK315990/
20. Beaunoyer E, Dupéré S, & Guitton MJ. COVID-19 and digital inequalities: Reciprocal impact and mitigation strategies. Computers in Human Behaviour. 2020;111:106424. Available from: https://doi.org/10.1016/j.chb.2020.106424
21. Andrew A, Cattan S, Costa Dias M et al. Inequalities in Children's Experiences of Home Learning during the COVID-19 lockdown in England. Fiscal Studies. 2020;41(3):653-683. Available from: https://doi.org/10.1111/1475-5890.12240
22. Rennard U & McAlister B. UK Standards for Public Involvement – One 'very different' year on. [Internet] NIHR; 2020; Available from: https://www.nihr.ac.uk/blog/uk-standards-for-public-involvement-one-very-different-year-on/26600 [Accessed on 3 February 2020]
23. Gray, LM, Wong-Wylie, G, Rempel, RG and Cook, K. Expanding Qualitative Research Interviewing Strategies: Zoom Video Communications. The Qualitative Report. 2020;25(5):1292-1301. Available from: https://search.proquest.com/docview/2405672296?pq-origsite=gscholar&fromopenview=true
24. Gibson A, Bardach SH and Pope ND. COVID-19 and the Digital Divide: Will Social Workers Help Bridge the Gap? Journal of Gerontological Social Work. 2020;63(6-7):671-673. Available from: https://doi.org/10.1080/01634372.2020.1772438
25. Martins Van Jaarsveld G. The Effects of COVID-19 Among the Elderly Population: A Case for Closing the Digital Divide. Frontiers in Psychiatry. 2020;11:577427. Available from: https://dx.doi.org/10.3389%2Ffpsyt.2020.577427
26. Mitchell UA, Chebli PG, Ruggiero L & Muramatsu N. The Digital Divide in Health-Related Technology Use: The Significance of Race/Ethnicity. The Gerantologist. 2019;59(1):6-14. Available from: https://doi.org/10.1093/geront/gny138
27. Newlands G, Lutz C, Tamo-Larrieux A et al. Innovation under pressure: Implications for data privacy during the COVID-19 pandemic. Big Data & Society. 2020;7(2) Available from: https://doi.org/10.1177%2F2053951720976680
28. Fletcher FE, Allen S, Vickers SM, Beavers T, Hamlin CM, Young-Foster D, Harris-Turner S, Erwin PC. COVID-19's impact on the African American community: a stakeholder engagement approach to increase public awareness through virtual town halls. Health Equity 2020;4(1): 320–325, Available from: doi.org/10.1089/heq.2020.0029.
29. Zhou L, Thieret R, Watzlaf V, Dealmeida D, Parmanto B. A Telehealth Privacy and Security Self-Assessment Questionnaire for Telehealth Providers: Development and Validation. Int J Tele Rehabil. 2019;11(1):3-14. Available from: doi.org/10.5195/ijt.2019.6276
30. Ortega P, Martinez G and Diamond L. Language and Health Equity during COVID-19: Lessons and Opportunities. Journal of Health Care for the Poor and Underserved. 2020:31(4);1530-1535. Available from: https://doi.org/10.1353/hpu.2020.0114
31. Emmel N, Hughes K, Greenhalgh J, Sales A. Accessing Socially Excluded People — Trust and the Gatekeeper in the Researcher-Participant Relationship. Sociological Research Online. 2007;12(2):43-55. doi:10.5153/sro.1512

32. Gatekeepers: The politics of community. [Internet] Co-operative Innovation Project; 2015. Available from: https://coopinnovation.files.wordpress.com/2015/12/gatekeepers-cip-final.pdf [Accessed 9th November 2020]

For peer review only

Appendix 1: Survey questions

Challenges and solutions: What kind of CEI is feasible in LMICs during COVID-19?

As an NIHR global health research fund holder we are inviting you to participate in this survey.

The aim of this survey is to capture what kind of CEI activities are feasible in LMICs under the current challenging global circumstances, including how much CEI activities have changed and been adjusted to the new situation. We would like to know,

- what CEI activities have been unaffected and remain ongoing,
- any challenges faced and
- any potentially new, innovative ways of communication with communities in LMICs.

This survey is purely for learning purposes to share knowledge and expertise across global health networks, both within the NIHR and beyond, and so we can support each other in these challenging times. We hope to highlight efficient communication pathways with communities in LMICs that might be taken forward after this pandemic as general practice.

The survey should take approximately 15-20 minutes to complete. If you are unable to complete the survey in one sitting, your responses will be saved and you can return later to complete it. If you would like to nominate another colleague e.g. the lead for CEI to complete the survey, please forward the survey URL link to them.

Before you consider completing the survey please read the information sheet which was circulated with the email invitation. Thank you.

Your participation

The information you provide is confidential and you will not be identified in the reporting of findings unless you give your permission. General information about you, your role and your institution will be requested to help us analyse the survey results.

Your participation is voluntary. It is an opportunity to help us understand the impact of COVID-19 on CEI in LMIC global health research.

If you would like to complete the survey, please check the box below as your consent to participation. You will not be able to continue to the main survey without doing so.

Thank you for your time and input. It is much appreciated.

If you have any queries, please contact Katie Porter at ceiglobalhealth@nihr.ac.uk.

For peer review only

**1. About you (individual completing the survey) and your associated institution**	
Name	
Job title	
Associated organisation/institution	
Funding body/ies for your research in LMICs	
Broad research area/s	
Please tell us what LMICs you are collaborating with for ongoing and new research	
Are you happy to be contacted for further questions relating to the information you have provided in this survey?	☐Yes, please provide email address:<br><br>☐No

**2. Overview: Ongoing research projects and CEI activities in LMICs**<br>When we refer to ongoing research in this survey, we mean research projects designed and started prior to the COVID-19 pandemic.<br>**If you have no ongoing research projects, please skip ahead to section 6.**	
Are any of your ongoing research projects currently going ahead?	☐Yes<br>☐No

If yes to above:

How many of your projects are currently going ahead with no effect on CEI activities despite COVID-19?	
How many of your projects are currently going ahead, but CEI activities had to be paused due to COVID-19?	
How many of your projects are currently going ahead, but CEI activities had to be amended due to COVID-19?	

**3. Overview: New research projects in LMICs**<br>When we refer to new research in this survey, we mean research projects designed and started after or in response to the onset of the COVID-19 pandemic, to be started within the next few months (whilst COVID-19 is still likely to be affecting research in LMICs)	
Have you planned and/or started any new research projects in LMICs since the start of the COVID-19 pandemic?	☐Yes<br>☐No
If yes, do any of these new research projects have CEI components planned in?	☐Yes<br>☐No
If yes, how many of these projects have CEI components planned in?	

For peer review only

**4. The next 2 sections will ask you to provide more details about the CEI activities with your ongoing and new research projects in LMICs. This is only relevant to you if you have previously stated that you have ongoing or new CEI activities of any kind.**<br><br>**If you have previously selected that ALL your CEI activities had to be paused and/or no new activities were planned, please select "No CEI activities are being done" from the drop down menu to skip to the end of the survey**
☐ I am undertaking CEI activities. I would like to continue the survey<br>☐ No CEI activities are being done

<table>
<tr><td colspan="2">5. We are interested to capture what kind of CEI activities are feasible under the current circumstances.
Please provide a summary of what CEI activities are currently ongoing and provide explanation of how they are being carried out. If they are linked to specific research projects only, please fill in those details first.
(Free text, bullet point answers OK.)</td></tr>
<tr><td rowspan="3">Research project</td><td>Name:</td></tr>
<tr><td>LMIC:</td></tr>
<tr><td>☐Ongoing
☐New</td></tr>
<tr><td>CEI activities
(free text)</td><td></td></tr>
</table>

**6. What challenges have you faced in the current climate when carrying out CEI activities and/or planning CEI activities for new studies?**<br>Please provide the main reasons for these challenges.<br>*(free text, bullet points OK)*

**6. Were you able to find any feasible solutions to the challenges faced or new, innovative ways of going about CEI in LMICs during this pandemic?**<br>For example: new, successful ways of communicating with patient/public partners (please provide details of involved groups and LMICs, if so).<br>**Please include lessons learnt and top tips or any promising ideas.**<br>*(free text, bullet points OK)*

For peer review only

**7. Are there any CEI components you designed due to COVID-19 that you might adapt for future research projects?**<br>**If yes, please describe these CEI components and explain why you would continue to use them.**<br>*(free text, bullet points OK)*

For peer review only

Appendix 2: Round table questions

We invited Network members to talk about their observations and reflections to date of the CEI activities in LMIC research that they are leading on, involved in or are aware of. The aim of this round table was to hear about members' understanding and experience of the impact of COVID-19 on CEI activities in LMICs.

- What CEI activities are currently taking place during the pandemic?
- How have the original planned CEI activities changed as a consequence of COVID-19?
- What is the impact of COVID-19 is on CEI activities in LMIC research?
- What activities have been paused or discontinued?
- What CEI activities have been unaffected?
- How have CEI activities been adapted/changed?
- In summary, what have been the main barriers and challenges?
- How did you overcome these challenges?
- Are the CEI challenges different to the normal ones faced outside of the pandemic?
- Have you explored any new or innovative ways to engage or involve relevant people or communities with both ongoing and new research?

For peer review only

Appendix 3: Survey participant funding bodies

Funding body	No. of projects
NIHR	27
Medical Research Council (MRC)	8
Wellcome Trust	5
UK Research and Innovation (UKRI)/Research England	3
Economic and Social Research Council (ESRC)	2
Department for International Development (DFID)	2
Gavi The Vaccine Alliance	1
GlaxoSmithKline	1
AstraZeneca	1
Save the Children	1
International AIDS Vaccine Initiative (IAVI)	1
World Bank	1
Norwegian Government	1
National Institutes of Health (NIH)	1
European Union (EU) Research and Innovation programme Horizon 2020	1
European & Developing Countries Clinical Trials Partnership (EDCTP)	1
Bill & Melinda Gates Foundation	1

For peer review only

World Health Organisation (WHO)	1
FAIRMED Foundation	1
European Commission (EC)	1
HIV Vaccine Trials Network (HVTN)	1
The Global Fund	1
Unknown	1

For peer review only

Standards for Reporting Qualitative Research (SRQR)*

http://www.equator-network.org/reporting-guidelines/srqr/

Title and abstract

	Page/line no(s).
**Title** - Concise description of the nature and topic of the study Identifying the study as qualitative or indicating the approach (e.g., ethnography, grounded theory) or data collection methods (e.g., interview, focus group) is recommended	p1
**Abstract** - Summary of key elements of the study using the abstract format of the intended publication; typically includes background, purpose, methods, results, and conclusions	p1

Introduction

**Problem formulation** - Description and significance of the problem/phenomenon studied; review of relevant theory and empirical work; problem statement	p2-3
**Purpose or research question** - Purpose of the study and specific objectives or questions	p2-3

Methods

**Qualitative approach and research paradigm** - Qualitative approach (e.g., ethnography, grounded theory, case study, phenomenology, narrative research) and guiding theory if appropriate; identifying the research paradigm (e.g., postpositivist, constructivist/ interpretivist) is also recommended; rationale**	p3-4
**Researcher characteristics and reflexivity** - Researchers' characteristics that may influence the research, including personal attributes, qualifications/experience, relationship with participants, assumptions, and/or presuppositions; potential or actual interaction between researchers' characteristics and the research questions, approach, methods, results, and/or transferability	p3
**Context** - Setting/site and salient contextual factors; rationale**	p3-4
**Sampling strategy** - How and why research participants, documents, or events were selected; criteria for deciding when no further sampling was necessary (e.g., sampling saturation); rationale**	p3-4
**Ethical issues pertaining to human subjects** - Documentation of approval by an appropriate ethics review board and participant consent, or explanation for lack thereof; other confidentiality and data security issues	p3

**Data collection methods** - Types of data collected; details of data collection procedures including (as appropriate) start and stop dates of data collection and analysis, iterative process, triangulation of sources/methods, and modification of procedures in response to evolving study findings; rationale**	p3-4

1

Data collection instruments and technologies - Description of instruments (e.g., interview guides, questionnaires) and devices (e.g., audio recorders) used for data collection; if/how the instrument(s) changed over the course of the study

**Units of study** - Number and relevant characteristics of participants, documents, or events included in the study; level of participation (could be reported in results)	p4<br>Appendix 1<br>Appendix 2
**Data processing** - Methods for processing data prior to and during analysis, including transcription, data entry, data management and security, verification of data integrity, data coding, and anonymization/de-identification of excerpts	p4
**Data analysis** - Process by which inferences, themes, etc., were identified and developed, including the researchers involved in data analysis; usually references a specific paradigm or approach; rationale**	p4
**Techniques to enhance trustworthiness** - Techniques to enhance trustworthiness and credibility of data analysis (e.g., member checking, audit trail, triangulation); rationale**	p4

Results/findings

**Synthesis and interpretation** - Main findings (e.g., interpretations, inferences, and themes); might include development of a theory or model, or integration with prior research or theory	p4-10
**Links to empirical data** - Evidence (e.g., quotes, field notes, text excerpts, photographs) to substantiate analytic findings	p4-10

Discussion

**Integration with prior work, implications, transferability, and contribution(s) to the field -** Short summary of main findings; explanation of how findings and conclusions connect to, support, elaborate on, or challenge conclusions of earlier scholarship; discussion of scope of application/generalizability; identification of unique contribution(s) to scholarship in a discipline or field	p10-14
**Limitations** - Trustworthiness and limitations of findings	p12

Other

**Conflicts of interest** - Potential sources of influence or perceived influence on study conduct and conclusions; how these were managed	p15
**Funding** - Sources of funding and other support; role of funders in data collection, interpretation, and reporting	p15

*The authors created the SRQR by searching the literature to identify guidelines, reporting standards, and critical appraisal criteria for qualitative research; reviewing the reference lists of retrieved sources; and contacting experts to gain feedback. The SRQR aims to improve the transparency of all aspects of qualitative research by providing clear standards for reporting qualitative research.
**The rationale should briefly discuss the justification for choosing that theory, approach, method, or technique rather than other options available, the assumptions and limitations implicit in those choices, and how those choices influence study conclusions and transferability. As appropriate, the rationale for several items might be discussed together.

Reference:

O'Brien BC, Harris IB, Beckman TJ, Reed DA, Cook DA. **Standards for reporting qualitative research: a synthesis of recommendations.** *Academic Medicine*, Vol. 89, No. 9 / Sept 2014 DOI: 10.1097/ACM.0000000000000388

BMJ Open

Challenges and solutions: Surveying researchers on what type of community engagement and involvement activities are feasible in low and middle income countries during the COVID-19 pandemic

Journal:	*BMJ Open*
Manuscript ID	bmjopen-2021-052135.R1
Article Type:	Original research
Date Submitted by the Author:	01-Sep-2021
Complete List of Authors:	Kroese, Karolin; University of Birmingham<br>Porter, Katie; University of Southampton<br>Surridge, Heidi; University of Southampton<br>Tembo, Doreen; University of Southampton
**Primary Subject Heading**:	Global health
Secondary Subject Heading:	Qualitative research, Research methods
Keywords:	COVID-19, STATISTICS & RESEARCH METHODS, INFECTIOUS DISEASES

SCHOLARONE™
Manuscripts

I, the Submitting Author has the right to grant and does grant on behalf of all authors of the Work (as defined in the below author licence), an exclusive licence and/or a non-exclusive licence for contributions from authors who are: i) UK Crown employees; ii) where BMJ has agreed a CC-BY licence shall apply, and/or iii) in accordance with the terms applicable for US Federal Government officers or employees acting as part of their official duties; on a worldwide, perpetual, irrevocable, royalty-free basis to BMJ Publishing Group Ltd ("BMJ") its licensees and where the relevant Journal is co-owned by BMJ to the co-owners of the Journal, to publish the Work in this journal and any other BMJ products and to exploit all rights, as set out in our licence.

The Submitting Author accepts and understands that any supply made under these terms is made by BMJ to the Submitting Author unless you are acting as an employee on behalf of your employer or a postgraduate student of an affiliated institution which is paying any applicable article publishing charge ("APC") for Open Access articles. Where the Submitting Author wishes to make the Work available on an Open Access basis (and intends to pay the relevant APC), the terms of reuse of such Open Access shall be governed by a Creative Commons licence – details of these licences and which Creative Commons licence will apply to this Work are set out in our licence referred to above.

Other than as permitted in any relevant BMJ Author's Self Archiving Policies, I confirm this Work has not been accepted for publication elsewhere, is not being considered for publication elsewhere and does not duplicate material already published. I confirm all authors consent to publication of this Work and authorise the granting of this licence.

For peer review only

Challenges and solutions: Surveying researchers on what type of community engagement and involvement activities are feasible in low and middle income countries during the COVID-19 pandemic

Authors: Dr Karolin Kroese [1], Katie Porter [2], Heidi Surridge [2], Dr Doreen Tembo [2]

Affiliations:

1. NIHR Global Health Research Unit on Global Surgery, University of Birmingham, Birmingham, UK
2. Wessex Institute, Faculty of Medicine, University of Southampton, Southampton, UK

Correspondence: Katie Porter, katie.porter@nihr.ac.uk

Word count (excluding title page, abstract, references, figures and tables): 5078 words

Abstract

Objectives: Measures to limit the spread of infection during the COVID-19 global pandemic have made engaging and involving members of the community in global health research more challenging. This research aimed to explore how global health researchers adapted to the imposed pandemic measures in low and middle income countries (LMICs) and how they overcame challenges to effective community engagement and involvement (CEI).

Design: A qualitative two-stage mixed methods study involving an online survey and a virtual round table.

Setting: The survey and round table were completed online.

Participants: Of 53 participants, 43 were LMIC or UK-based global health researchers and/or CEI professionals, and 10 worked for the National Institute for Health Research or UK Government's Department of Health and Social Care.

Outcome measures: This study aimed to capture data on: the number of CEI activities halted and adapted because of the COVID-19 pandemic; where CEI is possible; how it has been adapted; what the challenges and successes were; and the potential impact of adapted or halted CEI on global health research.

Results: Pandemic control measures forced the majority of researchers to stop or amend their planned CEI activities. Most face-to-face CEI activities were replaced with remote methods, such as online communication. Virtual engagement enabled researchers to maintain already established relationships with community members, but was less effective when developing new relationships or addressing challenges around the inclusion of marginalised community groups.

Conclusions: COVID-19 has highlighted the need for contingency planning and flexibility in CEI. The re-designing and adopting of remote methods has come with both advantages and disadvantages, and required new skills, access to technology, funding, reliable services and enthusiasm from stakeholders.

For peer review only - http://bmjopen.bmj.com/site/about/guidelines.xhtml

The methods suggested have the potential to augment or substitute previously preferred CEI activities. The effectiveness and impact of these remote CEI activities need to be assessed.

Article summary: Strengths and limitations of this study

- This study used a two-stage mixed method evaluation using an online survey and an international round table to capture the impact of the COVID-19 pandemic on community engagement and involvement activities.
- The participants represent a broad variety of LMICs across multiple continents, therefore providing a good overview of CEI activities across the board.
- The level of seniority and expertise of participants is varied, ensuring an accurate representation of CEI adaptation across the global health research network.
- Data was triangulated and coded to generate common emergent themes from the qualitative data to understand the main challenges and barriers research teams have faced, and how their community engagement and involvement activities have been adapted in global health research.
- Due to the sample size and timeframe when data was collected, this study only provides a snapshot of the community engagement and involvement activities being done in global health research early on in the pandemic (June/July 2020), but it provides an opportunity for shared learning and contingency planning in a unique situation.

Introduction

The development and expansion of the UK National Institute for Health Research (NIHR) and other UK-based funding bodies into the Global Health arena has necessitated a rethink of what is known as patient and public involvement (PPI) in the UK. UK-based global health researchers had to explore ways to involve community members in low and middle income countries (LMICs) where their research is taking place, using paradigms that are feasible, accepted and effective in a local context. In global health research, the involvement of public members is internationally more commonly referred to as community engagement [1], and within NIHR's global health research community as community engagement and involvement (CEI).

While there are some variations in the definition of CEI, the one used by the US Centres for Disease Control and Prevention summarises the essence of community engagement as: "…the process of working collaboratively with and through groups of people affiliated by geographic proximity, special interest, or similar situations to address issues affecting the well-being of those people" [2]. Community can broadly be defined as a minimum social unit who have a stake in the proposed research. This could include individuals, groups, organisations, government bodies, peer-groups or social networks, and those who can influence or who may be affected by the research [3]. Community involvement can therefore be defined as the community actively participating or being involved in any aspect of the research cycle [4].

Carrying out meaningful CEI within research is important as it reduces the potential for exploitation of communities and facilitates the implementation of health research [4]. Furthermore, involving

For peer review only - http://bmjopen.bmj.com/site/about/guidelines.xhtml

communities in LMICs in health research funded by high income countries (HICs) serves to address possible ethical concerns and cultural differences such as distrust and concerns about scientific colonialism [5]. If done properly, e.g. following the UNICEF standards [1], CEI can help ensure that benefits of research can be utilised by those in need, build on local capacity and support a true culture change in low resource settings by empowering people at grassroots level.

As COVID-19 developed into a global pandemic and countries went into lockdown, engaging and involving members of the public in research became far more challenging. In this paper we define 'lockdown' as large-scale physical distancing measures and movement restrictions [6], however we are aware that this term is specific to a UK context and not necessarily used globally. Similarly, 'post-lockdown periods' are not a unified, global point in time, and are varied in both severity and timeframes.

Solutions to involving the public in research during lockdown in the UK rapidly evolved around online communication platforms, however, this may not be feasible or poses additional challenges in LMICs. In LMICs, CEI has focused heavily on face-to-face communication due to limited access to technology and the internet. Traditionally face-to-face communication has been relied upon as a direct way to overcome barriers to involvement and is a cultural preference in some countries [7].

This project focused on investigating how CEI in low-resource settings developed and adapted to the restrictions imposed as a response to the COVID-19 pandemic. We aimed to understand the impact the restrictions had on CEI in LMICs and the main challenges researchers had to overcome. We highlight how CEI is being redesigned to address some of these barriers, explore the limitations with these solutions and discuss the implications for CEI.

Methods

We used a two-stage mixed method evaluation using an online survey and an international round table. Data was collected across June to early July 2020. Three of the authors work for the NIHR, but are not directly involved in the commissioning process, and so were able to use their networks to contact and involve NIHR-funded awardees and the NIHR CEI Global Health Advisory Network in this research.

Survey

An online survey was designed to capture the impact of COVID-19 on CEI activities, the main challenges and barriers research teams have faced, and how CEI activities have been adapted (see appendix 1). The survey captured quantitative data on demographics of participants and their research, as well as qualitative free text data on their CEI pandemic experiences. LMIC-based research team members, including public members and NIHR CEI professionals, reviewed the survey and contributed to its development.

For peer review only - http://bmjopen.bmj.com/site/about/guidelines.xhtml

The sampling used a combination of purposive and snowballing techniques [8]. The survey link, an invitation email and information sheet, were sent to all NIHR funded (and thus UK Aid funded) Global Health researchers and infrastructure awardees (n=70). The survey invitation was also posted on the Mesh Community Engagement Network website to access non-NIHR funded researchers. NIHR colleagues and networks shared the survey invitation with their own networks in the Global Health Research Community.

Round table

A round table discussion exploring the same topic was hosted as part of this research project. Participants were invited from the NIHR's Global Health CEI Advisory Network [9], with members based in the UK, as well as LMICs, all undertaking research with extensive CEI components. Participants therefore offered a wealth of experience and knowledge in undertaking CEI in different LMIC contexts. All members who took part had completed consent forms and received an information sheet prior to the round table. Three of the authors work regularly with this Network to help shape and inform NIHR's CEI portfolio, and so relationships with participants were already established.

Two of the authors facilitated the online round table (via Zoom). The discussion was guided by a series of questions agreed by the research team prior to the round table (appendix 2). Qualitative research notes from the round table were taken by the research team and circulated to participants for validation.

Data analysis

Using thematic data analysis, three individual members of the team analysed the triangulated survey and round table data. Collection and analysis of the quantitative data was supported by Microsoft Excel. NVivo software supported the analysis of the qualitative data. Themes were discussed by the research team and went through several iterations as discussions progressed. The resultant common emergent themes are described in the results section.

Patient and Public Involvement

As this research aimed to capture the researcher's perspective, with the primary target audience being global health researchers and funders, patients and community members were not specifically asked to be involved in the design and undertaking of this research. However, as part of the planning process, representative individuals from LMIC-based research teams, including members of the public and CEI professionals, were asked to review the questions to ensure relevant information on CEI activities was being captured. They were also asked to trial the online survey tool for usability and clarity prior to it being circulated.

Furthermore, when participating in the survey, lead researchers were encouraged in the invitation email to consult with their wider team prior to submitting their response. This included community members, where applicable and relevant. The collected data describes how research teams are engaging and involving community members in the research process.

For peer review only - http://bmjopen.bmj.com/site/about/guidelines.xhtml

This was a reactive piece of monitoring and evaluative work initiated by NIHR and an initial query raised by a global health CEI specialist (KK) with a primary purpose of understanding the impact of the pandemic on the research community working in LMICs. This in turn forms learning for the funder on changing needs of researchers.

Results

Survey participant demographics

Thirty-one (31) surveys were returned. Information on participant demographics can be seen in table 1. Of these 31, 27 (87%) were in receipt of NIHR funding, but many reported support from other funders (see appendix 3). Most participants reported on multiple research projects in different LMICs, in thematically diverse research areas. Participants' research was being conducted mainly on the African continent (61 out of 116 individual projects for this question, 53%), followed by Asia (39 individual projects, 33%) and South America (10 individual projects, 9%).

Round table participant demographics

Twenty-two (22) people took part in the round table: 7 (32%) were LMIC-based global health researchers, 5 (23%) UK-based global health researchers, and 10 (45%) UK-based NIHR and Department of Health and Social Care (DHSC) staff members. See table 1 for more information.

Table 1: Participant demographics

	Survey participants	Round table participants
Total number of participants	31 (100%)	22 (100%)
Where are they based?		
UK	22 (70%)	15 (68%)
Central America	1 (3%)	
North Africa	1 (3%)	
East Africa	2 (6%)	3 (14%)
West Africa	1 (3%)	
South Africa	1 (3%)	1 (5%)
South East Asia		2 (9%)
South Asia	2 (6%)	1 (5%)
Not given	1 (3%)	
Role		
CEI Lead/Manager	3 (10%)	2 (9%)
Project/Programme Manager	7 (23%)	
Professor or Associate Professor	8 (26%)	3 (14%)
Research Fellow	1 (3%)	
Consultant	1 (3%)	3 (14%)
Medical worker or officer	2 (6%)	
Research Assistant	2 (6%)	
Research Associate	2 (6%)	
Researcher		2 (9%)
PhD student	1 (3%)	

For peer review only - http://bmjopen.bmj.com/site/about/guidelines.xhtml

Lecturer	1 (3%)	1 (5%)
CEO	1 (3%)	
Director	1 (3%)	1 (5%)
NIHR/DHSC staff member		10 (45%)

Impact of COVID-19 restrictions on CEI activities in LMIC based research

All respondents stated that initially all their studies included CEI components, which ranged from consultative activities like community advisory boards/groups and focus groups to more involved activities such as co-production of study materials, or members of communities undertaking data collection.

Due to COVID-19, overall 83% of survey participants' planned CEI activities had to be amended or paused. Details on this can be found in Table 2 and 3.

Table 2: Unaffected, paused and amended CEI activities of survey participants. Please note there is overlap between the amended and paused responses.

**Participants with ongoing or partly ongoing research studies**	28 (90% of survey participants)	**No changes to any or most CEI activities**	5 (17% of participants with ongoing research studies)
		**Paused some or all CEI activities**	24 (86% of participants with ongoing research studies)
		**Amended some or all CEI activities**	22 (79% of participants with ongoing research studies)

Table 3: CEI activities being planned

**Participants currently planning new LMIC-based research studies**	14 (45% of all survey participants)
**New studies containing CEI components**	11 (78% of all new studies)

For peer review only

For peer review only - http://bmjopen.bmj.com/site/about/guidelines.xhtml

Reasons for halting CEI activities

Restriction of face-to-face interaction

During country-specific periods of lockdown, which included non-essential travel restrictions and social distancing measures, all face-to-face CEI activities had to be paused. Restrictions meant that researchers were unable to travel to different geographical areas, especially remote locations, for research purposes, such as providing interventions or collecting data.

> *"CEI which involved movement of researchers from one area to another [...] were also halted to avoid the spread of COVID-19. This has had a serious negative impact on CEI [...] the expected beneficiaries did not benefit anything from the CEI community dementia awareness raising planned activities." (Survey respondent 25)*

Local engagement activities throughout the research process were reported by many as involving regular face-to-face communication with researchers, multi-level government and organisational stakeholders, as well as community members. In Africa, many engagement activities are carried out face-to-face with community leaders and other representative bodies, as a culturally well-respected form of communication (*round table member 2*).

> *"We had planned to develop all materials through co-design and to undertake a series of focus groups for this purpose within India. However, we have been unable to complete this." (Survey respondent 4)*

> *"The team in Nepal were already heavily involved in a number of community awareness campaigns, using radio, tv, local schools, women's cooperatives etc. Due to Covid-19 these were all cancelled." (Survey respondent 3)*

Diversion of resources

In some countries, governments decided that all non-essential research should be halted. Others found that their research partners were diverted to other pandemic-related activities or research.

> *"In two of the three countries the ethical and regulatory authorities have ordered that all "non-essential" research activities are stopped. We have been able to continue our interventional clinical trials as they are deemed to be of direct importance to patient care, but the CEI activities are viewed as non-essential." (Survey respondent 23)*

> *"Due to the pandemic, and our respiratory specialty, our teams have been pulled to the front-line- while in key stages of completing our programme's milestones!" (Survey respondent 7)*

For peer review only - http://bmjopen.bmj.com/site/about/guidelines.xhtml

CEI solutions to pandemic measures

Remote methods of CEI

Due to pandemic restrictions, online methods replaced planned face-to-face activities. These have been highlighted as positive alternatives for one-way communication, such as sharing documents or videos for training and awareness raising. A key benefit is that resources can be shared widely and repeatedly with minimal effort, cost and time.

"We now have a video recording of all our training and don't hand out paper manuals... Everyone receives a copy of the training on a USB and is free to revisit it whenever they need to." (Survey respondent 10)

Researchers in the Occupied Palestinian Territories found that large scale online campaigns to raise awareness of burns studies has proven effective, but where materials could not be distributed online, they planned to combine their CEI activities, in this case health research awareness raising materials, with ongoing COVID-19 initiatives.

"Pamphlets and materials were prepared for a house-to-house community awareness campaign before COVID-19 lockdown. They [Ethiopia research team] are working with the Ministry of Health to see if they can combine their work on spreading COVID-19 messages which will be house-to-house [...] with burn prevention messages." (Survey respondent 3)

For two-way communication, researchers quickly adapted to using video calls (Zoom, Skype, GoToMeeting), phone, text, WhatsApp, WeChat, and similar apps. Online workshops and questionnaires were also introduced as participants adapted to the restrictions. Researchers pointed out that WhatsApp groups and Zoom meetings are helpful communication tools across borders and for communication between meetings. Some are keen to maintain communication via these platforms after country-specific pandemic restrictions are lifted, in addition to face-to-face meetings, to ensure continuity of contact with community stakeholders.

"As the groups have been engaged from the initial stages of the research they are keen to continue and provide input into the developing projects […] WhatsApp groups are the most useful method of communication for the CEI groups in the countries in which we work. The groups used this method between meetings, but also complete group meetings now using this platform." (Survey respondent 15)

"We have been pleasantly surprised by the relatively good internet access available to our researchers in Nepal who have been working from home. This is encouraging as it suggests

For peer review only - http://bmjopen.bmj.com/site/about/guidelines.xhtml

that domestic internet access is better and more widespread than we initially imagined and may mean that some CEI activity is potentially feasible." (Survey respondent 17)

Online communication methods were well received by young people in some LMICs. For example, in Nepal, Facebook groups were created to help young men discuss their mental health and wellbeing during lockdown (*round table member 6*).

Planning of post-lockdown CEI activities

The enforced period of restrictions allowed researchers to develop and adapt research and CEI plans for future post-pandemic research projects, whilst recognising that flexibility may be needed long-term.

"All activity has been delayed due to COVID-19 but we have been able to do all the planning and will be ready to do face-to-face work once restrictions in Uganda and Tanzania are over. Things like filming and getting groups of patients together has not been possible due to restrictions within the country." (Survey respondent 5)

Challenges with adapted CEI activities

Small group meetings post lockdown

In some countries, participants reported that small group gatherings were possible as restrictions began to ease. However, the extra precautions of personal protective equipment (PPE) and smaller community group meetings have higher resource implications for CEI.

"The inability to hold massive community meetings at halls during this time is really challenging [...] Currently, we have broken down our scale to less than 30 per meeting which means more meetings and outings than preferred." (Survey respondent 10)

A research team also reported that on return to the community, post-lockdown, wearing the necessary facemasks and PPE resulted in caution and mistrust as they were not easily identifiable (*round table member 7*).

Community and stakeholder availability and priorities

Research partners in LMICs have found themselves diverted to the pandemic response, whilst others have reduced CEI in order to keep their research sustainable. Furthermore, the pandemic restrictions resulted in community members having to prioritise basic needs of food, work and care for their families over engaging with researchers.

"As our partners are all clinicians with a focus on respiratory care, many of them are being pulled into the front line." (Survey respondent 7)

For peer review only - http://bmjopen.bmj.com/site/about/guidelines.xhtml

"Fear from getting infected so they refuse participation in research. Lockdown leaves them with few hours to respond to researchers, their priority is their children, food etc." (Survey respondent 11)

Inclusivity: Access to, and connectivity with, remote technology

Whilst remote means of communication have been the main CEI method during COVID-19, it has not been without its challenges and limitations. The use of digital platforms all rely on the individuals having access, funds and ability to use the technology and the internet.

In India, access to a smartphone was common but many could only access the internet on their devices at work, where free WiFi was provided (*survey respondent 4*). One research team working in sub-Saharan Africa noted that WhatsApp has enabled researchers to continue their CEI as even though computer access is rare, mobile phone access is common (*survey respondent 15*). Others working in Liberia and Nepal reported no or limited access to computer or mobile devices. Where devices were available, participants reported that larger-scale remote meetings, workshops or training were problematic due to unreliable internet connections. To surmount some of these issues respondents suggested that research teams could provide data allowances and technology for community stakeholders already involved with their research to ensure ongoing communication. Obviously, this has cost implications and may need to be costed into funding applications under CEI budget.

"The challenge of poor Internet connectivity often frustrated seamless virtual meetings with colleagues and stakeholders. We had to spend more funds purchasing alternative data service provider modems, and increased budget providing data for team members working from home." (Survey respondent 31)

"Communication is often challenging as a lot of the groups that we engage with have limited access to technology platforms- the digital divide." (Liberia - Survey respondent 27)

Teams who set up virtual groups using text-based platforms, such as WhatsApp, faced language and literacy barriers. One research group reported using voice notes as a way around this (*survey respondent 24*). Participants also noted that being able to see everyone's numbers on WhatsApp raised safeguarding concerns.

Remote communication was also thought to further marginalise some groups, for example in a study in Pakistan, husbands were often gatekeepers to women's phone access (*survey respondent 12*). Another team working with religious minority groups and in slum settlements found that many people, especially women and older people, had no internet access (*round table member 7*).

"Unable to have face to face contact since lockdown due to COVID-19 so using telephone but this can be difficult for some groups such as women in Pakistan who may only have

For peer review only - http://bmjopen.bmj.com/site/about/guidelines.xhtml

> *access to their husband's telephone[…] Could miss some of the most vulnerable people in the community by only having telephone communication." (Survey respondent 12)*

Many study participants reported that online and digital communication for CEI activities highlighted the social and economic inequalities within and between countries.

Quality of relationships built remotely

Some participants referred to remote communication as "second best" - an interim measure with concerns based on quality. Telephone and virtual meeting conversations were described as shorter, formal and less natural than face-to-face interactions. They were deemed tolerable for established relationships, but not ideal for making crucial stakeholder connections and establishing new relationships. Respondents therefore tended to focus on maintaining and developing relationships that were established before lockdown.

> *"Face to face communication is highly valued in Nepali society. Taking the trouble to visit someone in person demonstrates that you value them and their opinion, and is essential for building relationships." (Nepal – Survey respondent 17)*

> *"We have also used online Zoom webinars, which are useful in engaging people and groups, however, compared to face-to-face meetings, they lack the networking and 'connection' which face-to-face conferences and meetings have (...) this is very personal and valuable." (China – Survey respondent 7)*

Some research teams have reverted to online surveys and posting information, where previously these engagements would have been conducted face-to-face and hence interactively.

Discussion

Impact of COVID-19 on CEI

CEI activities are important for researchers in building and maintaining trust with local communities [10], and so the authoritarian nature of strict lockdowns and lack of information on COVID-19 in some LMICs has put this in jeopardy. As lockdown restrictions ease globally, researchers need to consider elevated levels of suspicion, especially among new contacts. The use of PPE by researchers in particular was identified as an aspect that created mistrust within the community as researchers were not easily identifiable, however this mistrust may also stem from a lack of access to PPE for community members. If researchers have access to PPE and the community does not this may create a power imbalance and potentially lead to feelings of mistrust.

A pandemic situation re-emphasizes the community's priorities; basic needs of access to food, work, health and safety are prioritised over involvement with research [11]. As a way to keep communities engaged, researchers could explore options to partner with others delivering community health

For peer review only - http://bmjopen.bmj.com/site/about/guidelines.xhtml

interventions, such as COVID-19 vaccination programmes. These grassroots community workers, if adequately resourced, may be able to deliver wider CEI activities alongside their own interventions. Funders also need to understand shifting community needs and priorities and potentially allow for re-prioritisation in projects in such circumstances. Also as research continues, project teams will need to consider the ethics of continuing CEI activities in relation to the needs of the community, especially as this pattern may continue as communities face prolonged impact of pandemic restrictions.

Impact of COVID-19 on research

The quality and impact of the amended CEI activities and impact on the research they support has yet to be investigated, especially when considering the limitations to remote CEI activities. It has been reported that CEI adaptations to pandemic measures have ultimately been inequitable and the needs of resource-poor and underrepresented communities were given little consideration [12]. The rapid response of many COVID-19 research projects has caused some to abandon regular engagement processes and focus on the voice of "professionals" [13]. In emergency pandemic situations, we need to ensure that the voice of the community and public should not be lost [14].

Reduced or poorer quality CEI may affect the outcomes of a research study, it's impact [1] and potentially opportunities for future funding. Some of the participants in this study paused CEI activities that were not feasible to do whilst restrictions were in place and continued with research activities that were. The effect of this has not yet been explored with the projects reported on here, however stopping CEI activities whilst moving other aspects of the research forward may imply that the community's role is not vital in the design and delivery of the research. It must be considered that CEI activities were paused as what was planned was no longer feasible and research teams were in the transition process of adapting their CEI and research activities to adhere to pandemic restrictions.

In periods where CEI activities must be paused, time can be used to plan and adapt future CEI activities. This time can also be used to develop the skills of the research team and community members, in particular online communication skills. Moving forward, there may also need to be contingency plans in place for managing CEI in areas with high prevalence of unpredictable communicable diseases. These contingency plans would need to include funding, access and support in the alternative means of communication and activities for all stakeholders involved in CEI. This is naturally an issue that reaches beyond CEI and research alone.

For peer review only - http://bmjopen.bmj.com/site/about/guidelines.xhtml

Pros and cons of virtual engagement

Good working relationships with community stakeholders are essential to ensure the value and quality of research [15, 16], however trusted relationships take time to develop. Building and maintaining meaningful relationships with the community are an important component of CEI [17], and this study has shown that pre-pandemic relationships may be sustained despite the lack of face-to-face engagement. Remote two-way communication methods were reported as useful in maintaining continuity of existing relationships during the pandemic and were already a well-established communication method.

Collaboration and co-production of research relies on the development of trusting relationships [6, 10, 17] and developing these relationships relies on regular, open communication with the community [18, 19]. Virtual CEI methods have been less effective in reaching out and engaging new communities, especially isolated vulnerable groups [20]. A feasible solution to better engage relevant community stakeholders suggested by LMIC researchers in this study is to utilise grassroots organisations and mobilise local civil society groups, which has been an effective way during COVID-19 to reach communities [21, 22]. Existing relationships can then be built upon which may also enable rapid response mechanisms, especially with marginalised communities.

Inclusivity concerns

The digital divide

Often, community members in LMICs have no or limited access to devices or the internet, and when they do, internet connection may be unstable or confined to certain areas. Thus, lockdown restrictions have meant that researchers struggled to reliably reach some community members. This means the most marginalised are even more likely to be excluded. This is an issue in HICs as well as LMICs, highlighting the need for non-technology based engagement [20]. Although participants suggested that researchers can pay for data allowances and technology, this raises questions around whether funders would be willing to cover these costs. Generally, remote platforms for CEI activities have differed from what was originally planned, and the pandemic has required researchers to be creative. Research teams need to think flexibly about what is feasible, and how various technologies and online tools can be used in the longer term. The sudden onset and fluctuating incidence of this pandemic and subsequent restrictions means ongoing uncertainty and contingency plans for CEI should be in place. Like researchers, funding bodies could consider room for flexibility for CEI activities proposed by the applicant, e.g. welcoming alternative and multiple scenario CEI activities for proposed research projects and allowing for re-allocation of funds within the budget.

Some digital video software might provide a solution for people who have access to a phone, but no internet connection, as people can dial into meetings via phone calls. It could also be more cost-

For peer review only - http://bmjopen.bmj.com/site/about/guidelines.xhtml

effective, accessible and increase reach and diversity [23, 24]. However, for community members to use the application and feel confident to contribute in meetings, training might be required. Depending on the capacity of the research team, this may pose an issue when trying to involve large numbers of community members.

Age may also be a factor as young people, particularly those in developing countries, who have access to gadgets or the internet may already be comfortable using these methods of communication [25, 26, 27]. Thus, engagement with this age group via online platforms may be easier for researchers compared to engaging older age-groups [27].

Technology also raises concerns of data privacy in LMICs and HICs alike and is a concern in PPI/CEI, as well as telemedicine [28, 29]. However, data privacy is not a new concern especially in healthcare [29]. An adequate solution for data privacy is yet to be found [28].

Close-knit communities often rely on 'word of mouth', where information is passed verbally, in-person to members of the community who do not have access to technology [30]. This approach should not be encouraged in pandemic situations and is prone to perpetuating inaccuracies through onward translation.

Research teams, as well as funders, will need to maximise their knowledge and creativity with virtual engagement whilst accepting that there may be limitations. Maintaining trust and relationships with community members is now more important than ever to ensure that they do not feel abandoned in times of crisis, to make research relevant and beneficial and to allow for involvement of key stakeholders when designing and implementing new studies remotely (for example, rapid response for COVID-19 studies). The use of digital tools is a way to do this, albeit perceived as not as inclusive or with the same depth as face-to-face communication. This may change with improving knowledge, familiarity and access. Remote communication also ensures ongoing transparency by keeping community stakeholders informed of research progress and providing opportunities to contribute at all stages. However, the impact on the quality of CEI undertaken remotely or via mixed approaches needs to be investigated.

Language and literacy barriers

Messaging apps were used for ongoing communication with CEI groups, but participants reported language and literacy barriers. Members of a messaging group may speak different dialects or languages depending on where they are based within the country - or across countries. The reach across borders via virtual platforms, poses the challenge of finding a common language. Where face-to-face engagement would usually take place with skilled translators, rapid and accurate translation is not an easy option in live virtual chats and meetings [31].

For peer review only

Marginalised communities

Participants reported that a common way to reach community members is via gatekeepers, who are often health workers or community leaders (for example, faith leaders or community elders). Accessing marginalised groups via gatekeepers is an established method for reaching a certain cohort of people based on, for example, location or faith [32]. However, participants noted that there can be limitations when accessing these communities via gatekeepers as they may restrict access to certain groups or individuals.

Another factor is the influence and power that gatekeepers often have over their communities. Some gatekeepers may be resistant to or sceptical of change and may use their influence over the community to sway their opinions [33, 34]. Restrictions on face-to-face meetings mean that this issue may be enhanced, due to researchers relying fully on gatekeepers communicating potentially complex studies to communities - potentially influencing audiences with biased views.

Limitations

The data for this research has come from a relatively small sample and was collected soon after COVID-19 had become a global pandemic, and so provides only a snapshot of the CEI activities being done in global health research. However, the authors saw this as an opportunity for shared learning in a unique situation and recommend that further research is done looking at the long-term impact of the COVID-19 pandemic on CEI activities.

Also in this study, the authors did not specifically invite community members to participate in the survey or round table. Given the resource constraints (mainly time-sensitivity and no allocated budget) and the potential barriers to inviting community members who are part of the research team (i.e., language, training, equal representation from all identified LMICs), this undertaking was not feasible for this piece of research. Participation of community members would have added a different perspective to our research which under the given circumstances was not feasible (as outlined in our paper, reaching out to community members during the pandemic is challenging), but would have also needed more resources, potential funding and manpower, which the authors did not have access to given that this was and is additional research alongside our normal workload. The authors recommend a community member perspective on the impact of the COVID-19 pandemic on CEI activities be explored further.

Conclusion

CEI adaptations were made in a fast-changing environment. There is much learning to be had for future similar localised and global situations, as well as during the ongoing COVID-19 pandemic. As countries gradually move towards a “new normal”, the way CEI is done may have changed forever as many researchers have experienced benefits from their adapted CEI activities. As research continues

For peer review only - http://bmjopen.bmj.com/site/about/guidelines.xhtml

in uncertain pandemic (or communicable disease) situations we recommend that research teams and funders should consider: using time effectively to plan for CEI activities when it is not currently possible; sharing relevant networks and resources; promoting acceptance and adoption of online technologies; adapting to the changing needs of the community; and developing contingency CEI plans to tackle the unexpected.

Declarations

Contributorship statement: KK, HS and KP contributed equally to the design and implementation of the research, to the analysis of the results and to the writing of the manuscript. DT contributed to, and commented on, this paper. The corresponding author attests that all listed authors meet authorship criteria and that no others meeting the criteria have been omitted.

Acknowledgements: We thank the UK National Institute for Health Research (NIHR) and Department of Health and Social Care staff who provided input into the paper based on their personal views. These included Gary Hickey (senior research manager, community engagement and involvement, UK NIHR), Aaronjay Tidball (global health research programme officer, science, research and evidence directorate, DHSC), Charlotte Seeley-Musgrave (global health research officer, global health research partnerships, DHSC), Patrick Wilson (head of global health communications and stakeholder engagement, UK NIHR), Sarah Puddicombe (assistant director, global health research, UK NIHR) and Jon Cole (assistant director, public involvement and external engagement, UK NIHR). We are also grateful to the NIHR CEI Global Health Advisory Network members who participated in the round table discussion.

Competing interests: We have read and understood BMJ's policy on declaration of interests and declare no conflicts of interest.

Ethics approval: We obtained full ethical approval for the research from the University of Southampton's Faculty of Medicine Ethics Committee (no. 53873).

Data sharing: All data relevant to the study are included in the article or uploaded as supplementary information. Additional data that support the findings of this study (free text data) are available from the corresponding author, Katie Porter, upon request.

Funding: This research was funded internally by the National Institute of Health Research (NIHR). Award/Grant number is not applicable.

References

1. Bedson J, Abramowitz S. Minimum Quality Standards and Indicators for Community Engagement. [Internet] UNICEF; 2020. Available from: https://www.unicef.org/mena/reports/community-engagement-standards [Accessed 9th November 2020]
2. McCloskey, DJ., McDonald, MA., Cook, J. et al. Chapter 1 Community Engagement: Definitions and Organizing Concepts from the Literature. In: Principles of Community Engagement. 2nd ed. Clinical and Translational Science Awards Consortium Community

For peer review only - http://bmjopen.bmj.com/site/about/guidelines.xhtml

Engagement Key Function Committee Task Force on the Principles of Community Engagement; 2011: 3-41 [Internet] Available from: https://www.atsdr.cdc.gov/communityengagement/pce_intro.html [Accessed 9th November 2020]

3. Council for International Organizations of Medical Sciences (CIOMS). International Ethical Guidelines for Health-related Research Involving Humans. 4th ed. Geneva; 2016: 25-27 [Internet] Available from: https://cioms.ch/wp-content/uploads/2017/01/WEB-CIOMS-EthicalGuidelines.pdf [Accessed 9th November 2020]
4. Musesengwa, R., Chimbari, M.J. Community engagement practices in Southern Africa: Review and thematic synthesis of studies done in Botswana, Zimbabwe and South Africa. Acta Trop 2017; 175: 20-30. Available from: http://dx.doi.org/10.1016/j.actatropica.2016.03.021
5. King, KF., Kolopack, P., Merritt, MW., and Lavery, JV. Community engagement and the human infrastructure of global health research. BMC Medical Ethics 2014; 15(84). Available from: https://doi.org/10.1186/1472-6939-15-84
6. WHO. Coronavirus Disease (COVID-19): Herd Immunity, Lockdowns And COVID-19. [online] 2020. Available at: https://www.who.int/news-room/q-a-detail/herd-immunity-lockdowns-and-covid-19 [Accessed 18 January 2021]
7. Tindana PO, De Vries J and Kamuya D. Ethical challenges in community engagement practices in research during the COVID-19 pandemic in Africa [version 1; peer review: 1 approved, 1 approved with reservations]. AAS Open Res 2020, 3:23. Available from: doi.org/10.12688/aasopenres.13084.1
8. Streeton, R, Cooker, M, and Campbell J. Researching the researchers: Using a snowballing technique. Nurse Researcher (through 2013). 2004;12(1):35-46. Available from: https://search.proquest.com/scholarly-journals/researching-researchers-using-snowballing/docview/200839960/se-2?accountid=13963
9. Mesh. 2021. NIHR Global Health CEI Advisory Network. Mesh. [online] Available at: <https://mesh.tghn.org/programme-hubs/nihr-global-health-ce-involvement/> [Accessed 1 February 2021].
10. Folayan, MO, Brown, B, Haire, B, Babalola, CP, Ndembi, N. Considerations for stakeholder engagement and COVID-19 related clinical trials' conduct in sub-Saharan Africa. Developing World Bioeth. 2020; 00: 1– 7. Available from: https://doi.org/10.1111/dewb.12283
11. Dahab, M, van Zandvoort, K, Flasche, S et al. COVID-19 control in low-income settings and displaced populations: what can realistically be done? Confl Health 2020; 14(54). Available from: https://doi.org/10.1186/s13031-020-00296-8
12. Cash R and Patel V. Has COVID-19 subverted global health? The Lancet. 2020; 395(10238): 1687-1688. Available from: https://doi.org/10.1016/S0140-6736(20)31089-8
13. Murphy E., Tierney E., Shé, Éidín N N., Killilea M., Donaghey C., Daly A., Roche M., Mac Loughlin D., Dinneen S.F., Panel P.I. COVID-19: Public and patient involvement, now more than ever. HRB Open Res. 2020;3:35. Available from: https://dx.doi.org/10.12688%2Fhrbopenres.13067.1
14. Marston, C, Renedo, A, and Miles S. Community participation is crucial in a pandemic. The Lancet. 2020;395(10238):1676-1678. Available from: https://doi.org/10.1016/S0140-6736(20)31054-0

15. Ryan MJ, Giles-Vernick T & Graham JE. Technologies of trust in epidemic response: openness, reflexivity and accountability during the 2014-2016 Ebola outbreak in West Africa. BMJ Global Health. 2019;4:e001272. Available from: http://dx.doi.org/10.1136/bmjgh-2018-001272
16. Woolf SH, Zimmerman E, Haley A & Krist AH. Authentic engagement of patients and communities can transform research, practice, and policy. Health Affairs. 2016;35(4):590-594. Available from: https://www.healthaffairs.org/doi/pdf/10.1377/hlthaff.2015.1512
17. Wilson, P, Mathie, E, Keenan, J, et al. ReseArch with Patient and Public invOlvement: a RealisT evaluation – the RAPPORT study. Southampton (UK): NIHR Journals Library; 2015 Sep. (Health Services and Delivery Research, No. 3.38.) Chapter 7, Discussion. Available from: https://www.ncbi.nlm.nih.gov/books/NBK315990/
18. Adhikari B, Pell C & Cheah PH. Community engagement and ethical global health research. Global Bioethics. 2020;31(1):1-12. Available from: https://www.tandfonline.com/doi/full/10.1080/11287462.2019.1703504
19. Tembo D, Hickey G, Montenegro C et al. Effective engagement and involvement with community stakeholders in the co-production of global health research. BMJ. 2021;372:n178. Available from: https://www.bmj.com/content/372/bmj.n178
20. Beaunoyer E, Dupéré S, & Guitton MJ. COVID-19 and digital inequalities: Reciprocal impact and mitigation strategies. Computers in Human Behaviour. 2020;111:106424. Available from: https://doi.org/10.1016/j.chb.2020.106424
21. Ratneswaren, A. The I in COVID: The importance of community and patient involvement in COVID-19 research. Clinical Medicine Journal. 2020; 20(4):e120-122. Available from: doi.org/10.7861/clinmed.2020-0173
22. Ekzayez A, al-Khalil M, Jasiem M, et al. COVID-19 response in northwest Syria: innovation and community engagement in a complex conflict. Journal of Public Health 2020; 42(3): 504-509, Available from: https://doi.org/10.1093/pubmed/fdaa068
23. Andrew A, Cattan S, Costa Dias M et al. Inequalities in Children's Experiences of Home Learning during the COVID-19 lockdown in England. Fiscal Studies. 2020;41(3):653-683. Available from: https://doi.org/10.1111/1475-5890.12240
24. Rennard U & McAlister B. UK Standards for Public Involvement – One 'very different' year on. [Internet] NIHR; 2020; Available from: https://www.nihr.ac.uk/blog/uk-standards-for-public-involvement-one-very-different-year-on/26600 [Accessed on 3 February 2020]
25. Gray, LM, Wong-Wylie, G, Rempel, RG and Cook, K. Expanding Qualitative Research Interviewing Strategies: Zoom Video Communications. The Qualitative Report. 2020;25(5):1292-1301. Available from: https://search.proquest.com/docview/2405672296?pq-origsite=gscholar&fromopenview=true
26. Gibson A, Bardach SH and Pope ND. COVID-19 and the Digital Divide: Will Social Workers Help Bridge the Gap? Journal of Gerontological Social Work. 2020;63(6-7):671-673. Available from: https://doi.org/10.1080/01634372.2020.1772438
27. Martins Van Jaarsveld G. The Effects of COVID-19 Among the Elderly Population: A Case for Closing the Digital Divide. Frontiers in Psychiatry. 2020;11:577427. Available from: https://dx.doi.org/10.3389%2Ffpsyt.2020.577427

For peer review only - http://bmjopen.bmj.com/site/about/guidelines.xhtml

28. Mitchell UA, Chebli PG, Ruggiero L & Muramatsu N. The Digital Divide in Health-Related Technology Use: The Significance of Race/Ethnicity. The Gerantologist. 2019;59(1):6-14. Available from: https://doi.org/10.1093/geront/gny138
29. Newlands G, Lutz C, Tamo-Larrieux A et al. Innovation under pressure: Implications for data privacy during the COVID-19 pandemic. Big Data & Society. 2020;7(2) Available from: https://doi.org/10.1177%2F2053951720976680
30. Fletcher FE, Allen S, Vickers SM, Beavers T, Hamlin CM, Young-Foster D, Harris-Turner S, Erwin PC. COVID-19's impact on the African American community: a stakeholder engagement approach to increase public awareness through virtual town halls. Health Equity 2020;4(1): 320–325, Available from: doi.org/10.1089/heq.2020.0029.
31. Zhou L, Thieret R, Watzlaf V, Dealmeida D, Parmanto B. A Telehealth Privacy and Security Self-Assessment Questionnaire for Telehealth Providers: Development and Validation. Int J Tele Rehabil. 2019;11(1):3-14. Available from: doi.org/10.5195/ijt.2019.6276
32. Ortega P, Martinez G and Diamond L. Language and Health Equity during COVID-19: Lessons and Opportunities. Journal of Health Care for the Poor and Underserved. 2020:31(4);1530-1535. Available from: https://doi.org/10.1353/hpu.2020.0114
33. Emmel N, Hughes K, Greenhalgh J, Sales A. Accessing Socially Excluded People — Trust and the Gatekeeper in the Researcher-Participant Relationship. Sociological Research Online. 2007;12(2):43-55. https://doi.org/10.5153%2Fsro.1512
34. Gatekeepers: The politics of community. [Internet] Co-operative Innovation Project; 2015. Available from: https://coopinnovation.files.wordpress.com/2015/12/gatekeepers-cip-final.pdf [Accessed 9th November 2020]

For peer review only

Appendix 1: Survey questions

Challenges and solutions: What kind of CEI is feasible in LMICs during COVID-19?

As an NIHR global health research fund holder we are inviting you to participate in this survey.

The aim of this survey is to capture what kind of CEI activities are feasible in LMICs under the current challenging global circumstances, including how much CEI activities have changed and been adjusted to the new situation. We would like to know,

- what CEI activities have been unaffected and remain ongoing,
- any challenges faced and
- any potentially new, innovative ways of communication with communities in LMICs.

This survey is purely for learning purposes to share knowledge and expertise across global health networks, both within the NIHR and beyond, and so we can support each other in these challenging times. We hope to highlight efficient communication pathways with communities in LMICs that might be taken forward after this pandemic as general practice.

The survey should take approximately 15-20 minutes to complete. If you are unable to complete the survey in one sitting, your responses will be saved and you can return later to complete it. If you would like to nominate another colleague e.g. the lead for CEI to complete the survey, please forward the survey URL link to them.

Before you consider completing the survey please read the information sheet which was circulated with the email invitation. Thank you.

Your participation

The information you provide is confidential and you will not be identified in the reporting of findings unless you give your permission. General information about you, your role and your institution will be requested to help us analyse the survey results.

Your participation is voluntary. It is an opportunity to help us understand the impact of COVID-19 on CEI in LMIC global health research.

If you would like to complete the survey, please check the box below as your consent to participation. You will not be able to continue to the main survey without doing so.

Thank you for your time and input. It is much appreciated.

If you have any queries, please contact Katie Porter at ceiglobalhealth@nihr.ac.uk.

For peer review only

1. **About you (individual completing the survey) and your associated institution**	
Name	
Job title	
Associated organisation/institution	
Funding body/ies for your research in LMICs	
Broad research area/s	
Please tell us what LMICs you are collaborating with for ongoing and new research	
Are you happy to be contacted for further questions relating to the information you have provided in this survey?	☐Yes, please provide email address:<br><br>☐No

2. **Overview: Ongoing research projects and CEI activities in LMICs**<br>When we refer to ongoing research in this survey, we mean research projects designed and started prior to the COVID-19 pandemic.<br>**If you have no ongoing research projects, please skip ahead to section 6.**	
Are any of your ongoing research projects currently going ahead?	☐Yes<br>☐No

If yes to above:

How many of your projects are currently going ahead with no effect on CEI activities despite COVID-19?	
How many of your projects are currently going ahead, but CEI activities had to be paused due to COVID-19?	
How many of your projects are currently going ahead, but CEI activities had to be amended due to COVID-19?	

3. **Overview: New research projects in LMICs**<br>When we refer to new research in this survey, we mean research projects designed and started after or in response to the onset of the COVID-19 pandemic, to be started within the next few months (whilst COVID-19 is still likely to be affecting research in LMICs)	
Have you planned and/or started any new research projects in LMICs since the start of the COVID-19 pandemic?	☐Yes<br>☐No
If yes, do any of these new research projects have CEI components planned in?	☐Yes<br>☐No
If yes, how many of these projects have CEI components planned in?	

For peer review only

**4. The next 2 sections will ask you to provide more details about the CEI activities with your ongoing and new research projects in LMICs. This is only relevant to you if you have previously stated that you have ongoing or new CEI activities of any kind.**<br><br>**If you have previously selected that ALL your CEI activities had to be paused and/or no new activities were planned, please select "No CEI activities are being done" from the drop down menu to skip to the end of the survey**	
☐ I am undertaking CEI activities. I would like to continue the survey<br>☐ No CEI activities are being done	

**5. We are interested to capture what kind of CEI activities are feasible under the current circumstances.**<br>**Please provide a summary of what CEI activities are currently ongoing and provide explanation of how they are being carried out. If they are linked to specific research projects only, please fill in those details first.**<br>*(Free text, bullet point answers OK.)*	
Research project	Name:
	LMIC:
	☐Ongoing<br>☐New
CEI activities<br>*(free text)*	

**6. What challenges have you faced in the current climate when carrying out CEI activities and/or planning CEI activities for new studies?**<br>Please provide the main reasons for these challenges.<br>*(free text, bullet points OK)*

**6. Were you able to find any feasible solutions to the challenges faced or new, innovative ways of going about CEI in LMICs during this pandemic?**<br>For example: new, successful ways of communicating with patient/public partners (please provide details of involved groups and LMICs, if so).<br>**Please include lessons learnt and top tips or any promising ideas.**<br>*(free text, bullet points OK)*

For peer review only

**7. Are there any CEI components you designed due to COVID-19 that you might adapt for future research projects?**<br>**If yes, please describe these CEI components and explain why you would continue to use them.**<br>*(free text, bullet points OK)*

For peer review only

Appendix 2: Round table questions

We invited Network members to talk about their observations and reflections to date of the CEI activities in LMIC research that they are leading on, involved in or are aware of. The aim of this round table was to hear about members' understanding and experience of the impact of COVID-19 on CEI activities in LMICs.

- What CEI activities are currently taking place during the pandemic?
- How have the original planned CEI activities changed as a consequence of COVID-19?
- What is the impact of COVID-19 is on CEI activities in LMIC research?
- What activities have been paused or discontinued?
- What CEI activities have been unaffected?
- How have CEI activities been adapted/changed?
- In summary, what have been the main barriers and challenges?
- How did you overcome these challenges?
- Are the CEI challenges different to the normal ones faced outside of the pandemic?
- Have you explored any new or innovative ways to engage or involve relevant people or communities with both ongoing and new research?

For peer review only

Appendix 3: Survey participant funding bodies

Funding body	No. of projects
NIHR	27
Medical Research Council (MRC)	8
Wellcome Trust	5
UK Research and Innovation (UKRI)/Research England	3
Economic and Social Research Council (ESRC)	2
Department for International Development (DFID)	2
Gavi The Vaccine Alliance	1
GlaxoSmithKline	1
AstraZeneca	1
Save the Children	1
International AIDS Vaccine Initiative (IAVI)	1
World Bank	1
Norwegian Government	1
National Institutes of Health (NIH)	1
European Union (EU) Research and Innovation programme Horizon 2020	1
European & Developing Countries Clinical Trials Partnership (EDCTP)	1
Bill & Melinda Gates Foundation	1

For peer review only

World Health Organisation (WHO)	1
FAIRMED Foundation	1
European Commission (EC)	1
HIV Vaccine Trials Network (HVTN)	1
The Global Fund	1
Unknown	1

For peer review only

Standards for Reporting Qualitative Research (SRQR)*

http://www.equator-network.org/reporting-guidelines/srqr/

Title and abstract

	Page/line no(s).
**Title** - Concise description of the nature and topic of the study Identifying the study as qualitative or indicating the approach (e.g., ethnography, grounded theory) or data collection methods (e.g., interview, focus group) is recommended	p1
**Abstract** - Summary of key elements of the study using the abstract format of the intended publication; typically includes background, purpose, methods, results, and conclusions	p1-2

Introduction

**Problem formulation** - Description and significance of the problem/phenomenon studied; review of relevant theory and empirical work; problem statement	p2-3
**Purpose or research question** - Purpose of the study and specific objectives or questions	p2-3

Methods

**Qualitative approach and research paradigm** - Qualitative approach (e.g., ethnography, grounded theory, case study, phenomenology, narrative research) and guiding theory if appropriate; identifying the research paradigm (e.g., postpositivist, constructivist/ interpretivist) is also recommended; rationale**	p3-4
**Researcher characteristics and reflexivity** - Researchers' characteristics that may influence the research, including personal attributes, qualifications/experience, relationship with participants, assumptions, and/or presuppositions; potential or actual interaction between researchers' characteristics and the research questions, approach, methods, results, and/or transferability	p3
**Context** - Setting/site and salient contextual factors; rationale**	p3-4
**Sampling strategy** - How and why research participants, documents, or events were selected; criteria for deciding when no further sampling was necessary (e.g., sampling saturation); rationale**	p3-4
**Ethical issues pertaining to human subjects** - Documentation of approval by an appropriate ethics review board and participant consent, or explanation for lack thereof; other confidentiality and data security issues	p4, p16

**Data collection methods** - Types of data collected; details of data collection procedures including (as appropriate) start and stop dates of data collection and analysis, iterative process, triangulation of sources/methods, and modification of procedures in response to evolving study findings; rationale**	p3-4

1

Data collection instruments and technologies - Description of instruments (e.g., interview guides, questionnaires) and devices (e.g., audio recorders) used for data collection; if/how the instrument(s) changed over the course of the study

**Units of study** - Number and relevant characteristics of participants, documents, or events included in the study; level of participation (could be reported in results)	p4, p5-6<br>Appendix 1<br>Appendix 2
**Data processing** - Methods for processing data prior to and during analysis, including transcription, data entry, data management and security, verification of data integrity, data coding, and anonymization/de-identification of excerpts	p4
**Data analysis** - Process by which inferences, themes, etc., were identified and developed, including the researchers involved in data analysis; usually references a specific paradigm or approach; rationale**	p4
**Techniques to enhance trustworthiness** - Techniques to enhance trustworthiness and credibility of data analysis (e.g., member checking, audit trail, triangulation); rationale**	p4

Results/findings

**Synthesis and interpretation** - Main findings (e.g., interpretations, inferences, and themes); might include development of a theory or model, or integration with prior research or theory	p5-11
**Links to empirical data** - Evidence (e.g., quotes, field notes, text excerpts, photographs) to substantiate analytic findings	p5-11

Discussion

**Integration with prior work, implications, transferability, and contribution(s) to the field -** Short summary of main findings; explanation of how findings and conclusions connect to, support, elaborate on, or challenge conclusions of earlier scholarship; discussion of scope of application/generalizability; identification of unique contribution(s) to scholarship in a discipline or field	p11-15
**Limitations** - Trustworthiness and limitations of findings	p15

Other

**Conflicts of interest** - Potential sources of influence or perceived influence on study conduct and conclusions; how these were managed	p16
**Funding** - Sources of funding and other support; role of funders in data collection, interpretation, and reporting	p16

*The authors created the SRQR by searching the literature to identify guidelines, reporting standards, and critical appraisal criteria for qualitative research; reviewing the reference lists of retrieved sources; and contacting experts to gain feedback. The SRQR aims to improve the transparency of all aspects of qualitative research by providing clear standards for reporting qualitative research.

**The rationale should briefly discuss the justification for choosing that theory, approach, method, or technique rather than other options available, the assumptions and limitations implicit in those choices, and how those choices influence study conclusions and transferability. As appropriate, the rationale for several items might be discussed together.

Reference:

O'Brien BC, Harris IB, Beckman TJ, Reed DA, Cook DA. **Standards for reporting qualitative research: a synthesis of recommendations.** *Academic Medicine*, Vol. 89, No. 9 / Sept 2014 DOI: 10.1097/ACM.0000000000000388
